# Oxidant-Induced Alterations in the Adipocyte Transcriptome: Role of the Na,K-ATPase Oxidant Amplification Loop

**DOI:** 10.3390/ijms21165923

**Published:** 2020-08-18

**Authors:** Komal Sodhi, James Denvir, Jiang Liu, Juan R. Sanabria, Yiliang Chen, Roy Silverstein, Zijian Xie, Nader G. Abraham, Joseph I. Shapiro

**Affiliations:** 1 Departments of Medicine, Surgery, and Biomedical Sciences, Joan C. Edwards School of Medicine, Marshall University, Huntington, WV 25755, USA; sodhi@marshall.edu (K.S.); denvir@marshall.edu (J.D.); liuj@marshall.edu (J.L.); sanabriaj@marshall.edu (J.R.S.); 2 Department of Medicine, Medical College of Wisconsin, Milwaukee, WI 53226, USA; Yiliang.Chen@bcw.edu (Y.C.); rsilverstein@mcw.edu (R.S.); 3 Departments of Medicine and Pharmacology, New York Medical College, Valhalla, NY 10595, USA; nader_abraham@nymc.edu

**Keywords:** chronic renal failure, oxidative stress, adipocytes, Na,K-ATPase, uremic cardiomyopathy, RNA sequencing

## Abstract

(1) Background: Recently we have noted that adipocyte specific expression of the peptide, NaKtide, which was developed to attenuate the Na,K-ATPase oxidant amplification loop, could ameliorate the phenotypical features of uremic cardiomyopathy. We performed this study to better characterize the cellular transcriptomes that are involved in various biological pathways associated with adipocyte function occurring with renal failure. (2) Methods: RNAseq was performed on the visceral adipose tissue of animals subjected to partial nephrectomy. Specific expression of NaKtide in adipocytes was achieved using an adiponectin promoter. To better understand the cause of gene expression changes in vivo, 3T3L1 adipocytes were exposed to indoxyl sulfate (IS) or oxidized low density lipoprotein (oxLDL), with and without pNaKtide (the cell permeant form of NaKtide). RNAseq was also performed on these samples. (3) Results: We noted a large number of adipocyte genes were altered in experimental renal failure. Adipocyte specific NaKtide expression reversed most of these abnormalities. High correlation with some cardiac specific phenotypical features was noted amongst groups of these genes. In the murine adipocytes, both IS and oxLDL induced similar pathway changes as were noted in vivo, and pNaKtide appeared to reverse these changes. Network analysis demonstrated tremendous similarities between the network revealed by gene expression analysis with IS compared with oxLDL, and the combined in vitro dataset was noted to also have considerable similarity to that seen in vivo with experimental renal failure. (4) Conclusions: This study suggests that the myriad of phenotypical features seen with experimental renal failure may be fundamentally linked to oxidant stress within adipocytes.

## 1. Introduction

Oxidant stress plays a key role in the development of renal-failure-associated cardiomyopathy (also known as uremic cardiomyopathy), both experimentally and clinically [1]. Recent in vitro and in vivo studies have established that adipocytes exposed to oxidative stress dysregulate expression of adipocytokines and bioactive mediators, which are able to stimulate more oxidative stress [2,3,4,5]. Through decades of studies, we and others have demonstrated Na,K-ATPase, a P-type ATPase, has cell signaling functions distinct from its better-known effects on cellular ion content [6,7,8]. In addition to well characterized ligands for Na,K-ATPase, we have found that reactive oxygen species (ROS) are also activators of Na,K-ATPase signaling [6]. This is achieved via carbonylation of the α1 subunit, which activates the Src with downstream modulation of signaling pathways, ultimately leading to further generation of ROS [9,10]. Previous studies have demonstrated that glucose oxidase induced H_2_O_2_ stimulated direct α1 subunit carbonylation of Pro222 of the actuator (A) domain and this was found to be involved in renal proximal tubule Na,K-ATPase signaling transduction [11]. Similarly, our previously published in vivo studies have also demonstrated increased α1 subunit protein carbonylation in visceral adipose tissue, under increased oxidative stress, which was accompanied by increased Na,K-ATPase signaling transduction and phosphorylation of Src and ERK expression [4,12,13]. Thus, ROS are not only generated from the Na,K-ATPase signaling cascade, but also promote it, creating a feed-forward oxidant amplification loop [14,15]. This observation led to the development of a peptide, NaKtide, derived from the α1 subunit of Na,K-ATPase, which interacts with the kinase domain of Src, inhibiting Na,K-ATPase signaling [4,9,10,13,16,17,18]. In light of understanding the exacerbation of oxidant stress in adipocytes under pathological setting, the implication of this feed-forward mechanism is specifically of great interest. Previously published studies from our labs have specifically shown that Na,K-ATPase signaling pathway is critical to the pathophysiology of several experimental models of disease including obesity [4,12], atherosclerosis [19], nonalcoholic steatohepatitis [19], aging [20], metabolic syndrome [21] and experimental uremic cardiomyopathy [22]. Most recently, we have shown that this adipocyte dysfunction is also central to mechanisms underlying the pathogenesis of uremia, including uremic cardiomyopathy [13]. Specifically, we identified that animals exposed to experimental uremia demonstrated cardiac phenotypical features consistent with uremic cardiomyopathy specifically abnormalities of systolic and diastolic function, cardiac hypertrophy and fibrosis along with evidence of systemic inflammation. Administration of a western diet augmented these changes whereas adipocyte specific expression of NaKtide ameliorated them. Our data indicated that the activation of adipocyte Na,K-ATPase signaling in this murine model of experimental uremic cardiomyopathy altered the adipocyte phenotype, induced mitochondrial dysfunction, and consequently, altered the systemic metabolic profile and cardiac phenotype [13].

Although inflammation and oxidative stress in adipose tissue are the key players in various diseases [23,24,25], the underlying biological processes are complex and not yet precisely characterized. Advancement in transcriptomics provides a remarkable opportunity to study functional implications of the genetic variability in adipose tissue under different pathological conditions. Oxidative stress triggers a coordinated transcriptional response in adipocytes that in turn regulate various molecular pathways associated with adipocyte function, inflammation and oxidative stress [26,27]. The trans-omic network analysis in adipocytes exposed to oxidative stress provides insight into the molecular level interactions, and thereby interpretation of the associated biochemical events and their regulation. Apart from transcriptomic analysis in adipocytes, recent studies have demonstrated alteration of signaling pathways in other cell type including aortic smooth muscle cells. As vascular smooth muscle cells (VSMCs) are involved in inflammatory responses, these studies elucidated differential expression of genes, specifically associated with vasculature changes, metabolic homeostasis, inflammatory and cellular oxidative signaling mechanisms [28,29]. Recent studies using RNA sequencing performed on adipose tissue of high fat diet (HFD) fed mice revealed the depot specific differential expression of genes involved in lipogenesis, adipogenesis, inflammation, endoplasmic reticulum (ER) stress, unfolded protein response (UPR), energy expenditure, fatty acid oxidation and oxidative phosphorylation [30]. The major pathways that were altered in HFD fed mice were integrin signaling, nuclear factor erythroid 2 – related factor 2 (NRF2)-mediated oxidative stress response, B cell receptor signaling, IL8 signaling, PI3K/AKT signaling, mTOR signaling, nerve growth factor (NGF) signaling, platelet derived growth factor (PDGF) signaling and peroxisome proliferator-activated receptor (PPAR) signaling. The early and late obesity-induced inflammatory gene profile of adipose tissue in HFD were also reported recently [26]. The major pathways found to have significant alterations were ECM receptor interaction, focal adhesion pathways, pathways linked to inflammation, including NF-κβ and TNFα signaling, chemokine signaling pathways and leukocyte trans endothelial migration pathways.

The various differentially expressed genes associated with inflammation in adipose tissue have been elucidated previously [27,31,32,33]. The major inflammatory genes like MCP-1, TNF-α, IL-6, IL-8 and IL-1B were found to be dysregulated under inflammatory condition in adipocytes. The cellular response to oxidative stress and its role in insulin resistance have been previously studied in 3T3-L1 adipocytes which demonstrate the role of major pathways including MAPK signaling, insulin signaling, TGF-β signaling, JAK/STAT signaling, adipocytokine signaling, Type II diabetes mellitus, cytokine receptor interaction and p53 signaling with significant alteration in genes [26,33]. Prior studies have demonstrated that under chronic renal failure, the retention of uremic toxins stimulate a systemic oxidative stress and inflammatory response in vivo [13,22,34]. The cumulative line of evidence also suggests a direct effect of uremic toxins, along with other bioactive ligands like oxidized LDL (oxLDL), in inducing oxidant stress and inflammation in adipocytes in vitro [2,3,35].

To better understand the molecular phenotypical changes that occur within adipocytes subjected to oxidant stress, in the present work we examined transcriptomic changes (RNAseq data analysis) in adipocytes derived from in vivo adipose tissues obtained in the context of experimental renal failure, with and without treatment with adipocyte specific expression of the NaKtide peptide known to ameliorate oxidant stress. To provide better context, this was supplemented with RNAseq analysis of the transcriptome of 3T3L1 pre-adipocytes programmed to differentiate into adipocytes that were subjected to either the putative uremic toxin, indoxyl sulfate (IS), or oxidant stress induced by exposure to oxLDL.

## 2. Results

### 2.1. In Vivo Findings

Experimental renal failure was induced by PNx, whereas sham mice were used as controls. Sham or PNx mice were transduced with adipocyte specific NaKtide or GFP (without NaKtide) expression using lentivirus, as described under Material and Methods. The changes in animal phenotype were as previously reported [13]. Briefly, experimental renal failure induced a hypertrophic, fibrotic cardiomyopathy which was exacerbated by concomitant western diet (WD) and ameliorated by adipocyte (but not skeletal muscle) specific NaKtide expression. In the current study, visceral fat was studied in only four groups (*n* = 6/group) corresponding to sham + GFP expression (Control), sham + NaKtide expression (NaKtide), PNx + GFP expression (PNx) and PNx + NaKtide expression (PNx + NaKtide).

**Figure 1:**
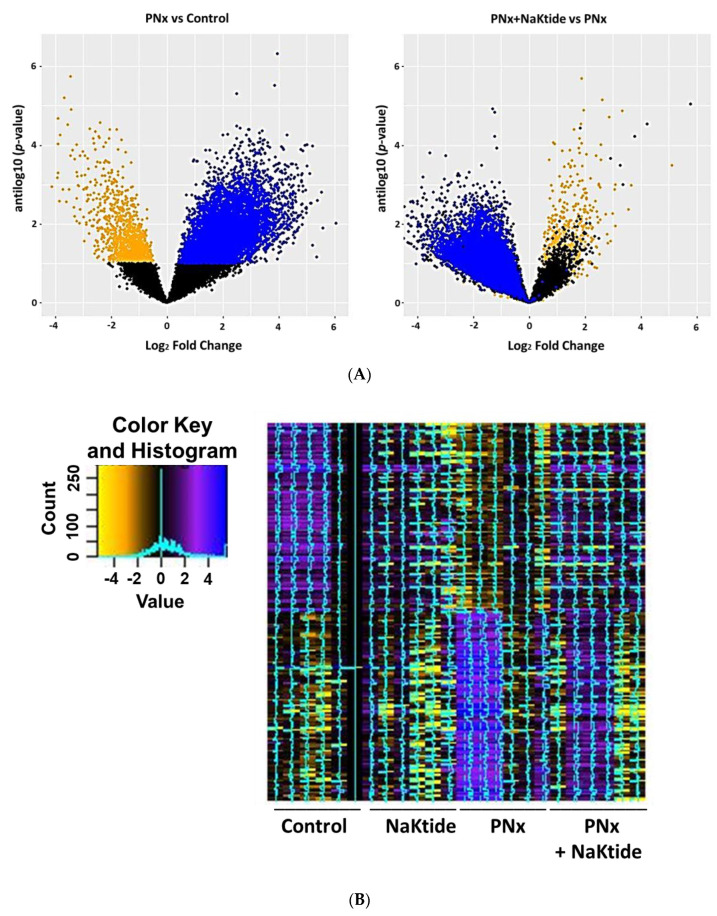

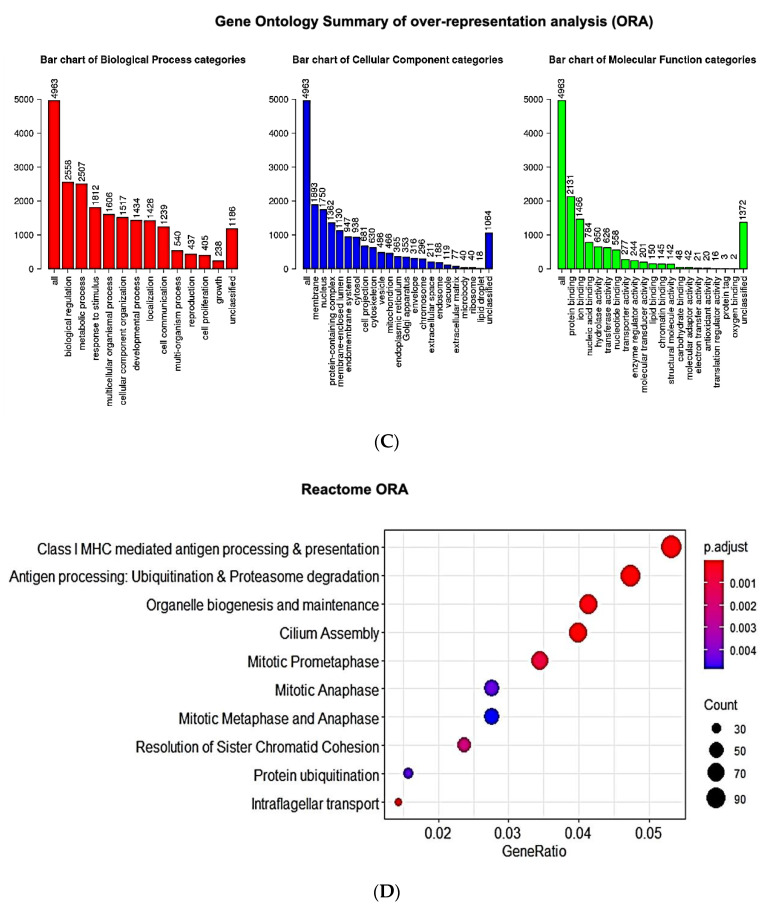
(**A**) Volcano plots of gene expression in PNx vs. Control (left panel) and PNx + NaKtide vs. PNx (right panel) plotting antilog of unadjusted *p*-value on y-axis vs. log_2_ Fold Change on x-axis. Genes downregulated (unadjusted *p*-value of <0.1) by PNx colored orange and genes upregulated (unadjusted *p*-value of <0.1) by PNx colored blue. We note that addition of NaKtide expression moved upregulated genes down and downregulated genes up. (**B**) Heat map of gene expression in top 100 up and 100 downregulated genes with PNx. Color coding based on log_2_ Fold Change with legend shown below. We note little difference between NaKtide and Control, but the addition of NaKtide to PNx appears to normalize both up and downregulated gene expression. (**C**) Gene Ontology summary of over representation analysis (ORA) in PNx mice model. Gene ontology annotation of biological processes, cellular components and molecular function categories. (**D**) Reactome ORA of differentially expressed genes in PNx mice model. The scatter dot plot of reactome enrichment representing the number of differentially expressed genes enriched in GO terms. *p*-value and gene ratio (number of differentially expressed genes in GO term)/(total number of genes in GO term) are shown in the plot. Larger circles indicate more enriched genes.

RNAseq analysis was performed following elimination of any gene which had <11 total counts in the 24 samples, in order to reduce the effect of genes which had very low expression. This resulted in a gene expression set containing 16,057 genes. Of these, 725 were noted to be downregulated and 4766 upregulated with an unadjusted *p*-value of 0.05. Using a Benjamini–Hochberg (BH)-adjusted *p*-value of 0.1, there were 5876 genes downregulated and 1148 genes upregulated. These results are shown by volcano plots in Figure 1A for the gene set comparing PNx and Control (left panel) and PNx + NaKtide vs. PNx (right panel). Interestingly, transduction of NaKtide in the adipose tissue tends to reverse changes in the gene expression, induced by PNx. This is further illustrated by a heat map of gene expression of the top 100 down and 100 upregulated genes shown in the sham and PNx animals ± adipose NaKtide expression (Figure 1B).

When we perform over-expression analysis, we note a number of pathways that could be identified. Using a false discovery rate (BH adjusted *p*-value) threshold of 10%, the gene-ontology changes with dataset limited to 4963 differentially expressed ontology categories are shown in Figure 1C. Furthermore, restricting ourselves to an unadjusted *p*-value of <0.10 (as in Figure 1A) using Reactome database, we also found a number of pathways which were differentially affected. Of interest, class I MHC mediated antigen processing as well as ubiquitination and protein processing were identified with relatively high confidence. A dot plot of the reactome enrichment is shown in Figure 1D. Examining Kyoto Encyclopedia of Genes and Genomes (KEGG) pathways with the same over expression analysis, we also have a number of pathways that were noted, only some of which were confirmed on the gene set enrichment analysis (GSEA) analysis also performed on the entire dataset (vida infra). Of interest because they were identified in both analyses, pathways related to ubiquitin proteolysis, cell cycle and protein processing appeared to be upregulated by experimental renal failure (Figure S1) and normalized by concomitant adipocyte specific NaKtide expression (Figure S2A–D).

The GSEA analysis also showed 115 KEGG pathways that were dysregulated by the experimental renal failure, all with an unadjusted *p*-value of <0.10. Whereas many of these pathways were related to the intermediary metabolism, there were also significant positives in signaling processes associated to the proteasome, mTOR, Hedgehog, p53, NOTCH, adipokine, PPAR, phosphoinositol, VEGF, and TGFβ signaling, peroxisome and hypertrophic cardiomyopathy. A summary of these pathways and their respective scores is provided in Table S1.

**Figure 2:**
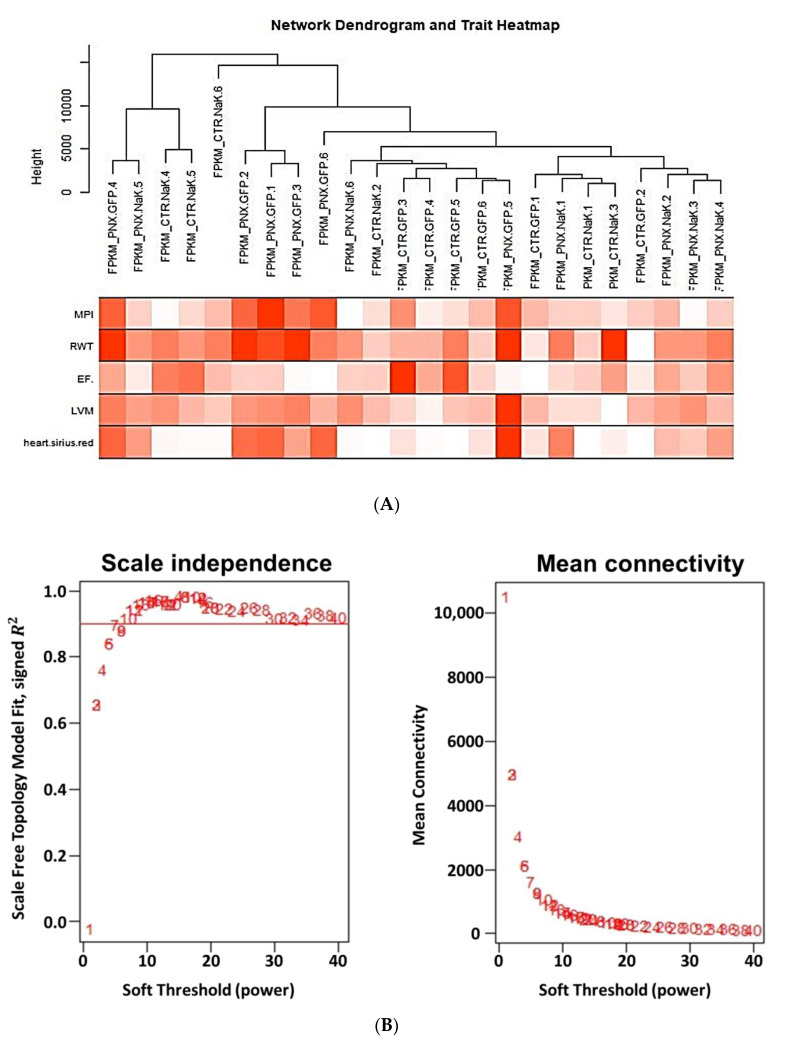

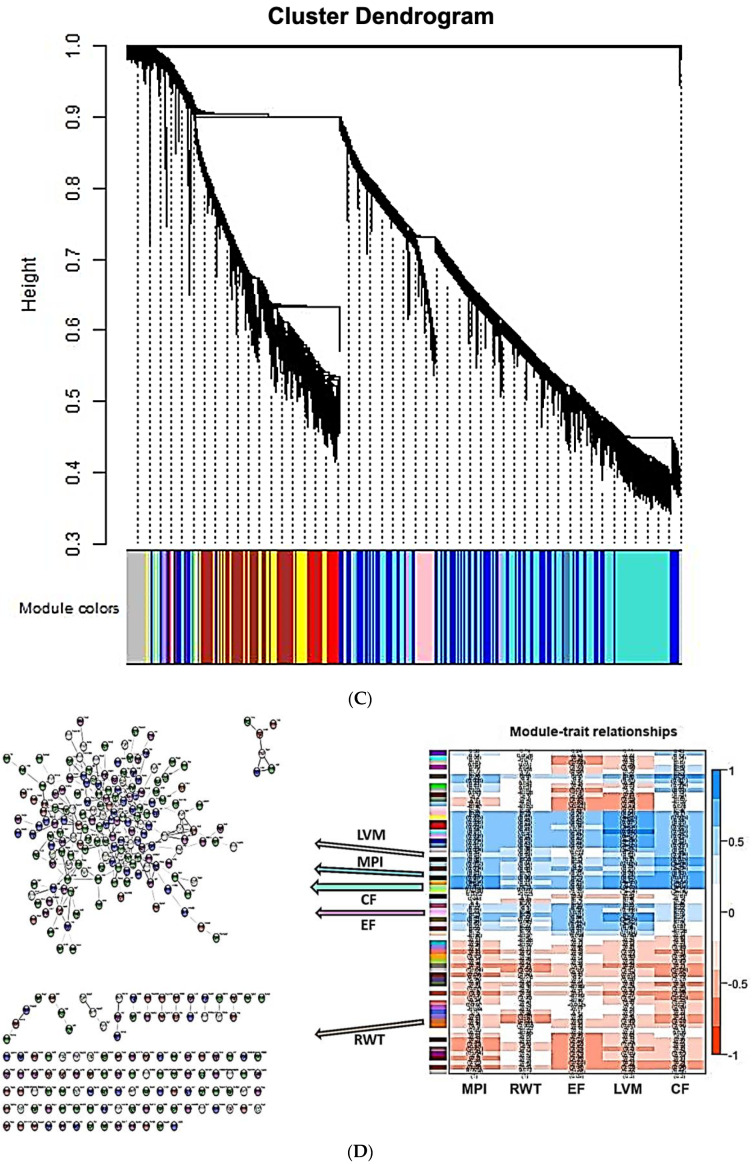
(**A**) Network dendrogram and trait heat map. Hierarchical clustering of treatment groups that summarize the modules found in the clustering analysis. Branches of the dendrogram cluster together into treatment groups that are positively correlated. Heat map displays correlation between different parameters analyzed. (**B**) Scale independence and mean connectivity as a function of soft threshold in PNx model. Based on these data, we chose a soft threshold (power) of 20 to construct the network(s) described in subsequent figures. (**C**) Dendrogram and group assignments produced from network generation on gene expression derived from in vivo experiment. Network produced using R package WGCNA where data on dendrogram represents distance metric (1-Pearson coefficient). Genes clustered according to a topological overlap metric into modules; assigned modules are colored at the bottom, gray genes are unassigned to a module. (**D**) Module-trait relationships of different parameters analyzed and major pathways associated with cardiac phenotype. Each row in the table (right panel) corresponds to different gene groupings, and each column to selected cardiac phenotypical features. Based on the highest correlations with these 5 phenotypical features (myocardial performance index (MPI), relative wall thickness (RWT), ejection fraction (EF), left ventricular mass (LVM) and cardiac fibrosis (CF)), five groups of genes were further analyzed for ORA against the KEGG database (left panel).

**Figure 3:**
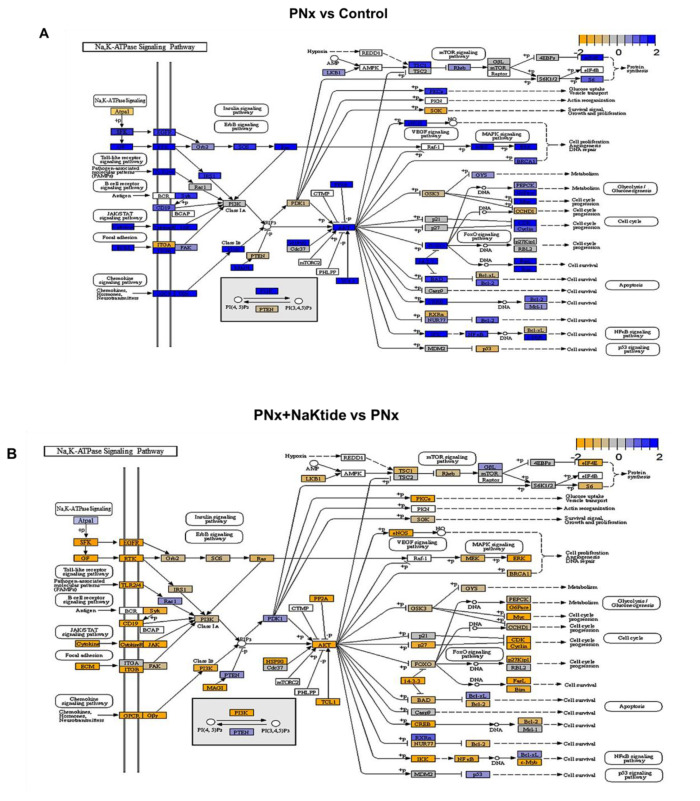

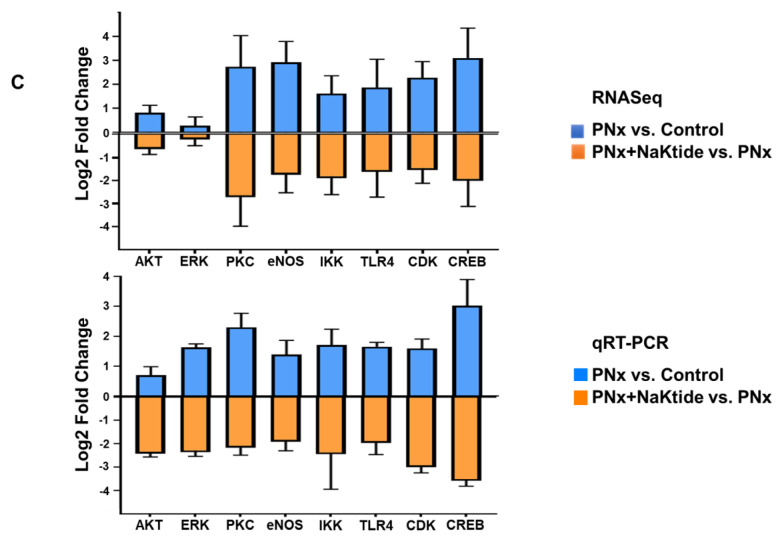
KEGG pathway analysis and validation of RNAseq data in visceral adipose tissue of PNx mice model with alteration of key genes associated with Na,K-ATPase signaling pathway. (**A**) PNx vs. Control (**B**) PNx + NaKtide vs. PNx. NaKtide administration to PNx appears to normalize both up and downregulated gene expression. Blue represents genes upregulated and orange represents genes downregulated. (**C**) Comparative gene expression analysis was done for eight representative genes selected from Na,K-ATPase signaling pathway from transcriptomic data and qRT-PCR. Results are expressed as log_2_ values of the fold change.

Using the topological approach, we found that the specific experiments segregated based on gene expression mostly along the lines of the experimental group assignments, with PNx having 3 out of 6 members separated out and the remaining three experiments mixed among other dendrogram cuts (Figure 2A). This was in agreement with the heat maps that were generated in these in vivo experiments (Figure 1B and Figure 2D) where these three experiments showed dramatically different gene expression patterns compared with other members of this experimental group as well as all other experiments. These three experimental animals had quite pronounced phenotypical features (Figure 2A). The power analysis suggested a soft threshold of 20 as suitable for network construction (Figure 2B), consistent with the analysis of the in vitro dataset (vida infra, Figure S8). The generation of a cluster dendrogram allowed for the identification of a number of gene groups which were found to have different degrees of correlation with in vivo phenotypical features (Figure 2C). In concordance with the phenotypical changes observed in our recently published study [13], the present transcriptomic profile showed a strong correlation with the characteristic features of experimental uremic cardiomyopathy including oxidative, inflammatory and mitochondrial changes concomitant with the dysregulation of adipose tissue Na,K-ATPase signaling. Specifically, the groups of genes that exhibited the highest absolute correlation with the 5 selected phenotypical features (myocardial performance index (MPI), relative wall thickness (RWT), ejection fraction (EF), left ventricular mass (LVM) and cardiac fibrosis) were then characterized with ORA (Figure 2D) and subsequently mapped with protein-protein interactions using the STRING database [36] and heat maps detailing selected gene expression in these different groups (Figure 2D and Figure S3A–E). When the KEGG pathway was studied, we found differential expression of various genes involved in Na,K-ATPase signaling pathway, as shown in Figure 3A,B. The validation of these findings was done using qRT-PCR. The expression levels of differentially expressed genes related to Na,K-ATPase signaling pathway including AKT, ERK, PKC, eNOS, IKK, TLR4, CDK and CREB were evaluated in the visceral adipose tissue of PNx mice. Our results from qRT-PCR were in concordance with the transcriptomic analysis as there was significant upregulation of these genes in PNx which was reverted by NaKtide transduction (Figure 3C).

### 2.2. In Vitro Findings

To further examine the molecular mechanisms, we performed experiments on 3T3L1 pre-adipocytes induced to adipogenesis. These cells were exposed to oxLDL to promote oxidant stress and IS to simulate experimental uremia, as well as concomitant treatment with pNaKtide. Phenotypical changes in these cells were also reported previously [2,37].

**Figure 4:**
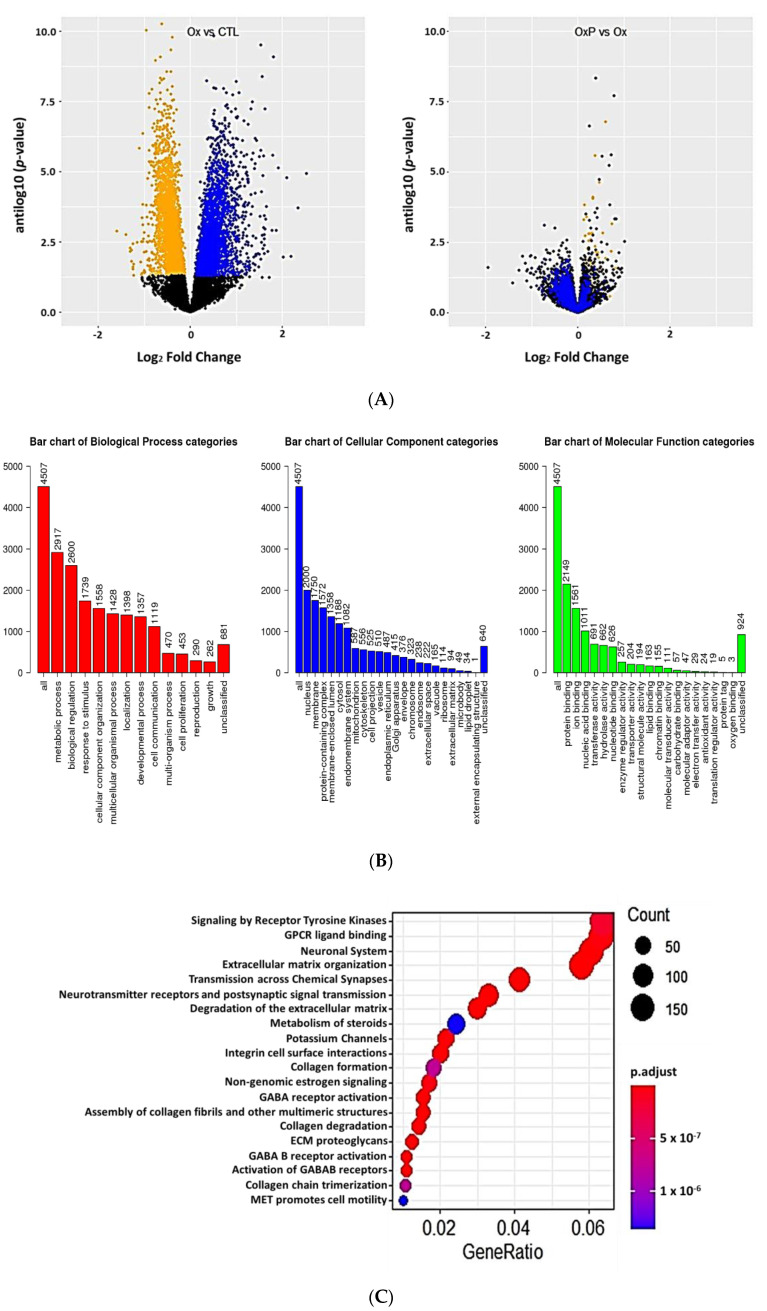
(**A**) Volcano plots of gene expression in adipocytes exposed to oxLDL (Ox) vs. control (CTL) (left panel) and oxLDL+ pNaKtide (OxP) vs. oxLDL (Ox) expression (right panel) plotting antilog of unadjusted *p*-value on y-axis vs. log_2_ Fold Change on x-axis. Genes downregulated (unadjusted *p*-value < 0.1) by oxLDL colored orange and genes upregulated (unadjusted *p*-value of <0.1) by oxLDL colored blue. We note that addition of pNaKtide expression moved upregulated genes down and downregulated genes up. (**B**) Gene Ontology summary of over representation analysis (ORA) in oxLDL treated murine adipocytes. Gene ontology annotation of biological processes, cellular components and molecular function categories. (**C**) Reactome ORA of differentially expressed genes in oxLDL treated murine adipocytes. The scatter dot plot of reactome enrichment representing the number of differentially expressed genes enriched in GO terms.

**Figure 5:**
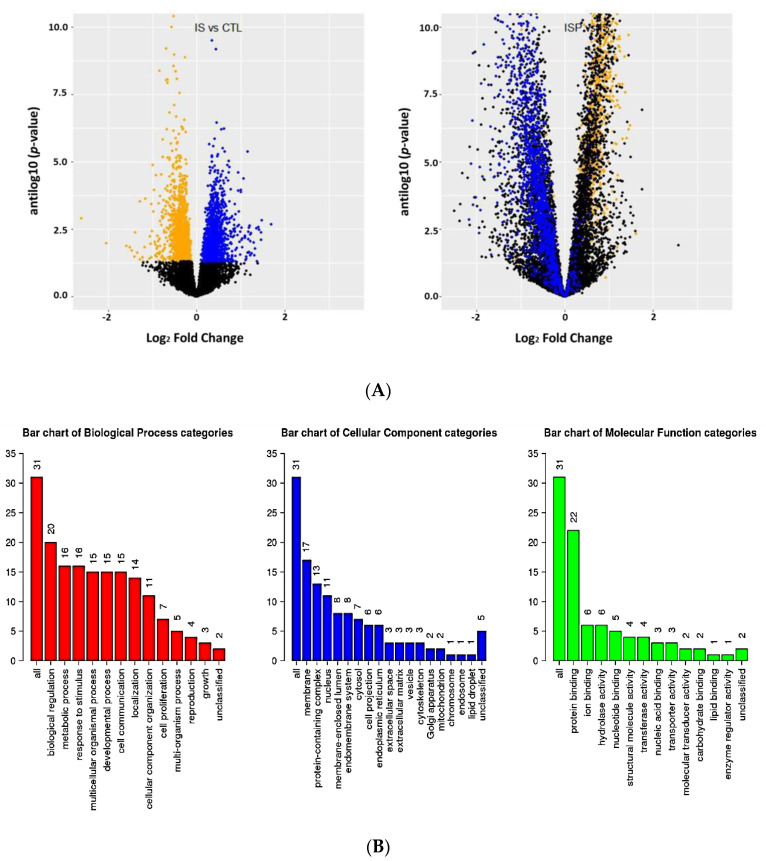
(**A**) Volcano plot of gene expression in adipocytes exposed to IS vs. control (CTL) (left panel) and IS+ pNaKtide (ISP) vs. IS expression (right panel) plotting antilog of unadjusted *p*-value on y-axis vs. log_2_ Fold Change on x-axis. Genes downregulated (unadjusted *p*-value of <0.1) by IS colored orange and genes upregulated (unadjusted *p*-value of <0.1) by IS colored blue. We note that addition of pNaKtide expression moved upregulated genes down and downregulated genes up. (**B**) Gene Ontology summary of over representation analysis (ORA) in IS treated murine adipocytes. Gene ontology annotation of biological processes, cellular components and molecular function categories.

Notably, 11,379 genes were expressed over the threshold of 14 counts in 30 samples. Among these, 3353 genes were under and 3042 genes were over-expressed in response to treatment with oxLDL using a BH-adjusted *p*-value of 0.10 (Figure 4A). When cells were treated with IS, 1894 genes were under and 1958 genes were over-expressed using a BH-adjusted *p*-value of 0.10 again (Figure 5A). For both oxLDL and IS, concomitant treatment with pNaKtide appeared to normalize gene expression (Figure 4A and Figure 5A).

Looking at the simple ORA with the Reactome dataset, oxLDL produced a large set (4507) of differentially expressed gene ontology processes (Figure 4B). The Reactome dataset showed particular enrichment in tyrosine kinase signaling and extracellular matrix pathways (Figure 4C). When the KEGG dataset was queried, we found several upregulated pathways of interest including the ubiquitin mediated proteolysis pathway (Figure S4A) which was normalized by pNaKtide treatment (Figure S4B).

When we performed similar analysis of IS, the effects on gene expression seemed quite similar to that observed with oxLDL as illustrated in the volcano plot of Figure 5A. However, surprisingly, we identified far fewer pathways by simple overexpression analysis compared to our findings with oxLDL. With gene ontology, only 31 pathways were noted, and with the Reactome dataset query, no pathways were identified (Figure 5B). Querying the KEGG pathway dataset, lipolysis, ECM-receptor interaction and protein processing in the endoplasmic reticulum were notably increased (Figure S5A). Again, concomitant pNaKtide treatment ameliorated the changes induced by IS as evidenced for the ECM-receptor interaction in Figure S5B.

**Figure 6:**
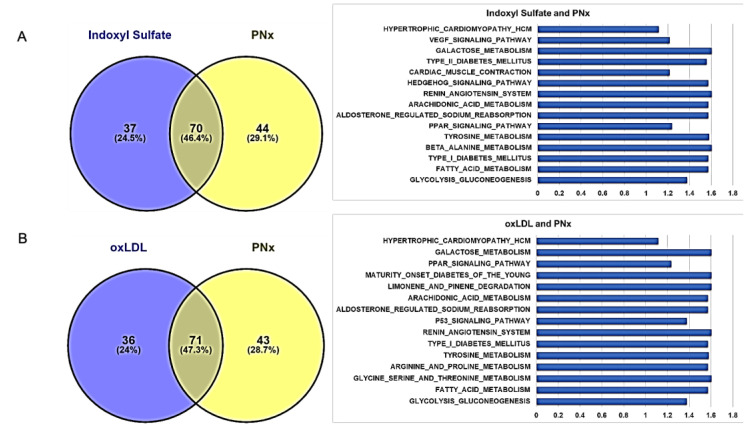

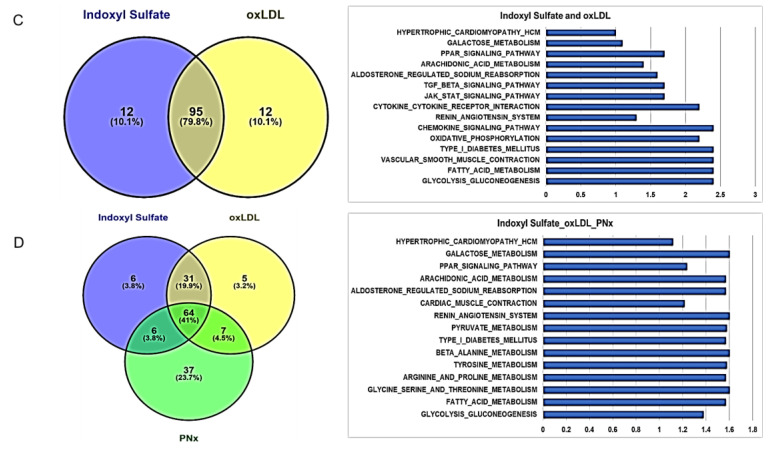
Pathway enrichment analysis using genes differentially expressed in vitro and in vivo in response to oxidative stress. The in vitro (oxLDL and IS treatment) and in vivo (PNx mouse model) response to oxidative stress differs in pathway enrichment, but some pathways overlap. The Venn diagram depicts the overlap of all enriched pathways among in vitro and in vivo, with selected common pathways (15 most relevant) and their BH-adjusted *p*-values depicted adjacent to the Venn diagram. Enriched pathways with little relevance to adipocyte biology have been omitted for clarity. Overlap of common pathways between (**A**) IS treatment and PNx mouse model, (**B**) oxLDL treatment and PNx mouse model, (**C**) IS and oxLDL treatment, and (**D**) in vitro (IS and oxLDL treatment) and in vivo (PNx mouse model).

**Figure 7:**
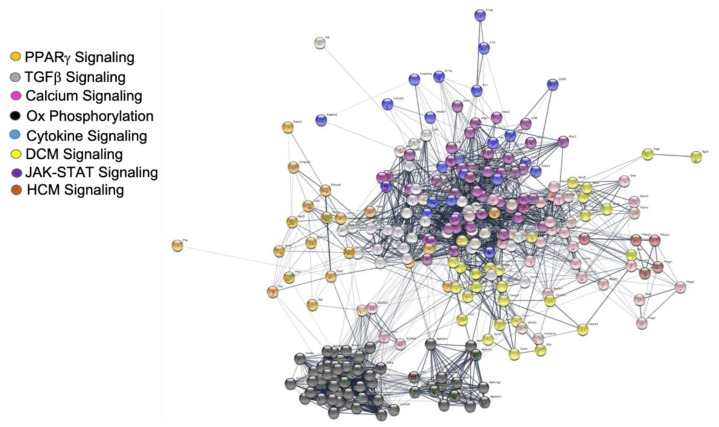
Network diagram associated with major pathways by GSEA altered in vitro. Color coding based on log_2_ Fold Change with legend shown above. All genes were identified in KEGG pathways and are therefore associated with other genes in the STRING database.

When GSEA was performed utilizing both the gene name and log_2_ fold change from control, we observed that 97 and 106 KEGG gene pathways were differentially regulated in the oxLDL and IS groups, respectively, all with unadjusted *p*–values of <0.1. These are summarized in Tables S2 and S3, for oxLDL and IS, respectively. The overlap between the in vivo and IS group was 70 pathways (Figure 6A) and between the in vivo and oxLDL group it was 71 (Figure 6B). The oxLDL and IS shared 95 out of 97 pathways identified for the oxLDL group (Figure 6C). The overlap between all three groups was 64 shared pathways (Figure 6D). This is shown graphically in Figure 6. From the shared in vitro pathways, we chose to focus on eight specific pathways whose protein–protein interactions are detailed in Figure 7. We also show the heat maps of genes involved in these pathways in Supplemental Figure S7A–H. We observed that both IS and oxLDL induced similar degrees of up and downregulation, both of which were normalized by concomitant pNaKtide treatment in these gene sets. We also note that when comparing pNaKtide alone to the control group, there were few differences in gene expression.

Although other differences certainly exist between oxLDL and IS treated cells, it is evident that one of the differences is that IS treated cells do not accumulate lipid well whereas oxLDL cells have greater lipid content than control 3T3L1 pre-adipocytes subjected to adipogenic media. Interestingly, although animals with experimental renal failure have metabolic syndrome which worsens with a western diet [38,39,40], these animals do not gain weight on this diet. Ergo, it is possible that one of the seven pathways identified in IS treated cells and adipose tissues from animals with experimental renal failure not seen in oxLDL treated cells may explain a shift in this phenotypical feature. Specifically, these pathways include the proteasome, p53 signaling pathway, retinol metabolism, TCA cycle, drug metabolism and other enzymes, intestinal immune network and folate biosynthesis (Figure S7A). However, when one examines the detailed heat maps of these specific gene expression patterns (Figure S7B–H), it appears that experimental noise is a more likely explanation for the differences between IS and oxLDL rather than a true difference in pathway activation or inhibition.

As indicated earlier, concomitant treatment of 3T3L1 cells with pNaKtide reversed most of the gene expression changes induced by either oxLDL or IS. However, pNaKtide alone resulted in very little change in gene expression compared to baseline in the cells not exposed to either oxLDL or IS. This can be seen in the collection of heat maps performed on the major signaling pathways that were differentially regulated in all three sets of experimental samples (Figures S3A–E, S6A–H and S7B–H). This was, of course, evident in the top 100 up and downregulated genes seen from the adipose tissue from animals with experimental renal failure (Figure 1B).

**Figure 8:**
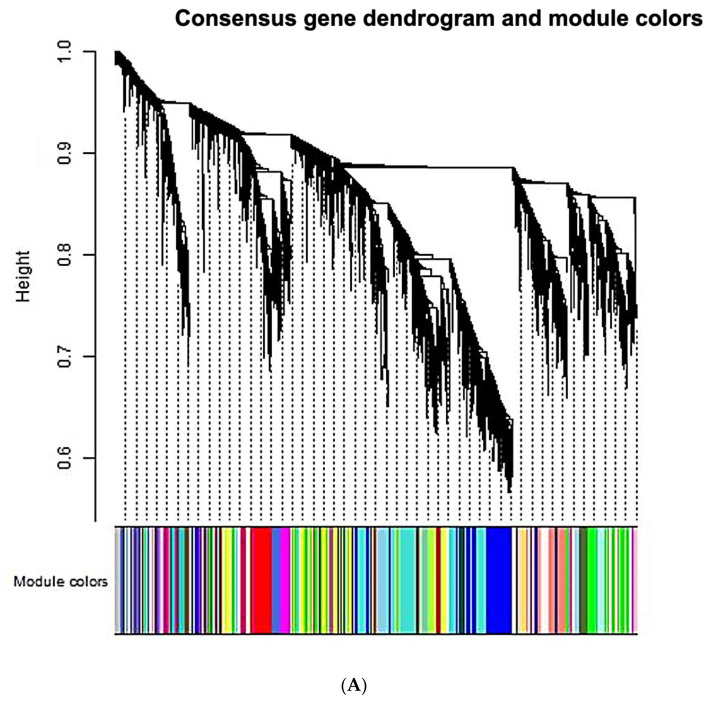

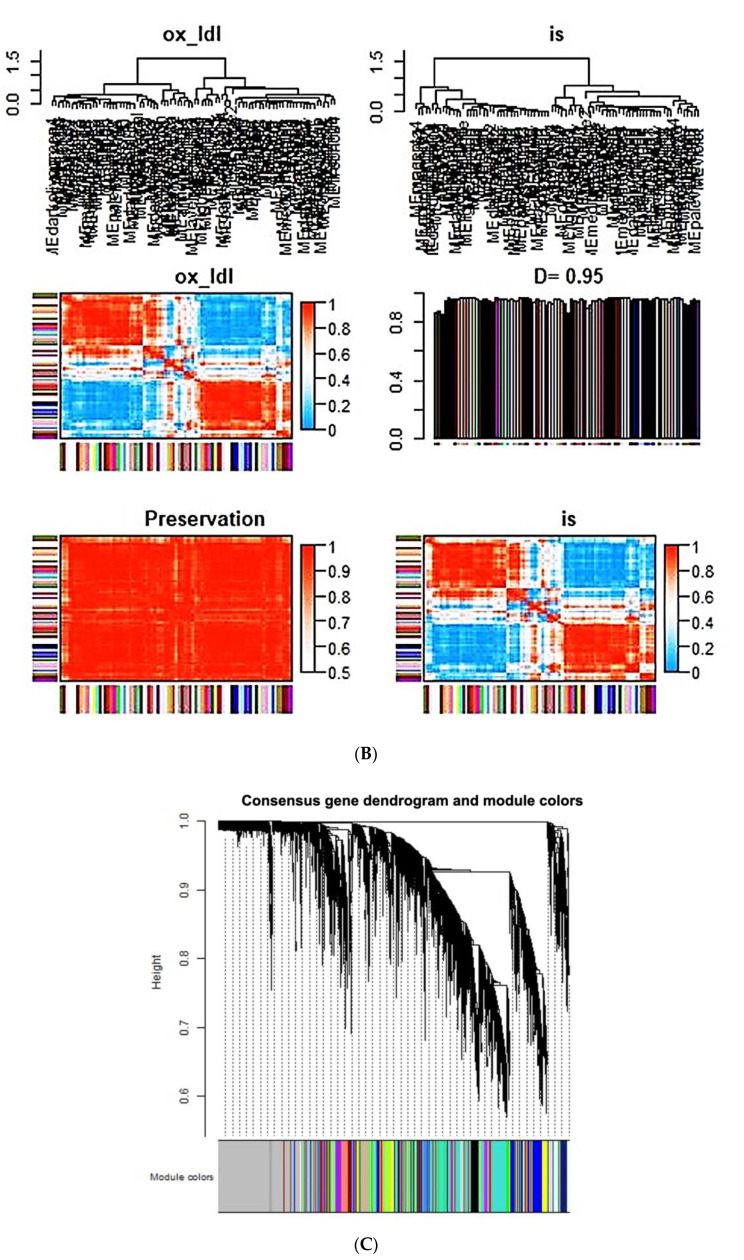

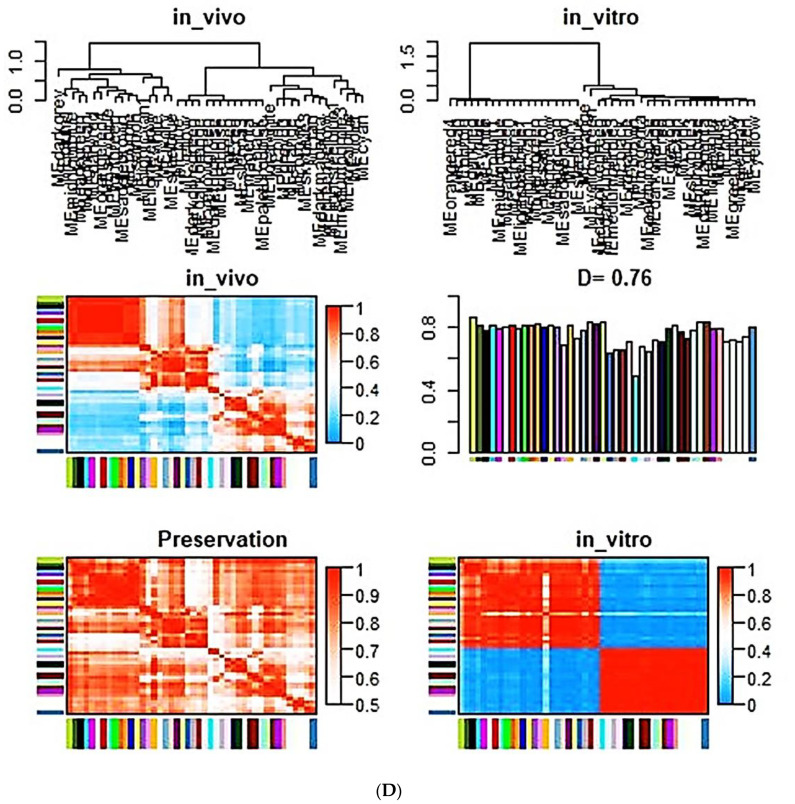
(**A**) Consensus network from in vitro experiments involving exposure to IS or oxLDL. Gene dendrogram obtained by average linkage hierarchical clustering for in vitro (IS and oxLDL) treatments. Gene expression similarity was determined using a pair-wise weighted correlation metric, and clustered according to a topological overlap metric into modules; assigned modules are colored at the bottom, gray genes are unassigned to a module. (**B**) Summary plot of consensus eigengene networks and their differential analysis from IS and oxLDL datasets. Top panels show clustering of consensus eigengenes in IS and oxLDL groups. Next, heat maps show high (red) and low (or negative, green) adjacency. Preservation heat map is 1-absolute difference of the eigengene networks in the two sets. Bar plot shows mean preservation of adjacency for each eigengene to other eigengenes with a D value calculated as the arithmetic mean of these measurements. (**C**) Consensus network from in vivo (PNx model) and in vitro experiments (both IS and oxLDL datasets). Gene dendrogram obtained by average linkage hierarchical clustering for in vivo and in vitro experiments. Gene expression similarity was determined using a pair-wise weighted correlation metric, and clustered according to a topological overlap metric into modules; assigned modules are colored at the bottom, gray genes are unassigned to a module. (**D**) Summary plot of consensus eigengene networks and their differential analysis from in vivo and in vitro (both IS and oxLDL) datasets. Top panels show clustering of consensus eigengenes in the two groups. Next, heat maps show high (red) and low (or negative, green) adjacency. Preservation heat map is 1-absolute difference of the eigengene networks in the two sets. Bar plot shows mean preservation of adjacency for each eigengene to other eigengenes with a D value calculated as the arithmetic mean of these measurements.

When we separated the in vitro set into an IS and oxLDL set, we noted that a soft threshold (power) of 20 allowed for a plateau in topological measurements (Figure S8). We noted the consensus gene dendrogram indicated strong similarity between the datasets (Figure 8A). This was confirmed by the groupings of the gene groups, the comparison of eigengene and group module characteristics with an overall similarity of 0.95 (Figure 8B). Comparing the combined in vitro set with the in vivo data previously discussed, similarities were also noted on the consensus dendrogram along with the eigengene and group module characteristics (Figure 8C). We note that overall similarity was 0.76 which, although still high, but clearly less than the in vitro response of adipocytes to IS and oxLDL (Figure 8D).

## 3. Discussion

We have recently reported that adipocyte-specific expression of NaKtide can ameliorate many aspects of experimental uremia including systemic oxidant stress, systemic and local cytokine elevations as well as the morphological, biochemical and functional alterations that are characteristic of uremic cardiomyopathy. With this background, we identified a large number of alterations in gene expression by RNAseq performed on RNA isolated from the visceral adipose tissue of mice with experimental renal failure using ORA and GSEA methodologies. Tremendous similarities were noted in gene expression changes induced by PNx in vivo compared with either IS or oxLDL treatment on cultured adipocytes in vitro. Most of these alterations were ameliorated by a blockade of the adipocyte Na,K-ATPase oxidant amplification loop by adipocyte specific expression of NaKtide in vivo or the pNaKtide treatment in 3T3L1 adipocytes in vitro. The in vivo changes in gene expression were highly correlated with a number of in vivo phenotypical features which we had previously reported [13]. Marked similarities in the patterns of gene dysregulation were noted by employing topological strategies, comparing the in vivo vs. in vitro datasets or between IS and oxLDL perturbations in the 3T3-L1 adipocytes.

Our findings with oxidative stress were in concordance with previous studies in murine HFD models or 3T3-L1 adipocytes, which reported consistent changes in gene expression which were associated with changes in adipocyte function [26,27,30,41]. As noted in the present study, several biological processes and signaling pathways were modulated in response to both oxLDL and IS in vitro and to experimental renal failure in vivo. In particular, marked changes in PPARγ signaling were noted in both in vitro and in vivo models as has been noted by Kim and coworkers [42]. Recent reports have demonstrated altered gene regulation of mediators involved in PPAR signaling in 3T3-L1 cells under oxidative stress conditions [26,43,44]. Similarly, large scale dysregulation of genes in response to oxidative stress in all three models were noted in pathways related to lipid metabolism, mitochondrial biogenesis and anti-oxidant defense as well as the JAK-STAT, TGFβ and cytokine signaling pathways. The bioactive mediators involved in the regulation of these pathways have been previously shown to stimulate oxidative stress and inflammation under diseased conditions with their role in alteration of adipocyte phenotype [45,46,47,48]. Similar findings were recently reported in genome wide transcriptomic analysis in 3T3-L1 adipocytes which demonstrated enrichment of these KEGG pathways in early and late response to oxidative stress and insulin resistance [26]. Oxidative phosphorylation is implicated in several biological and cellular processes including oxidative stress, mitochondrial metabolism, fatty acid metabolism, lipid homeostasis, fatty tissue inflammation and adipose tissue function [49,50,51]. A recent study reported enrichment of these important metabolic pathways in the white adipose tissue of mice fed a high fat diet [26,52]. These findings were in concordance with our in vitro and in vivo transcriptomic analyses, which caused differentially expressed genes in oxidative phosphorylation pathway under uremia or oxidative stress. In the cardiovascular system, oxidative stress stimulates pathways associated with cardiac hypertrophy that leads to pathological remodeling of the myocardium, impaired systolic function, fibroblast stimulation and the activation of metalloproteinases, ultimately resulting in chronic heart failure [53]. The oxidative stress and inflammation associated adipocyte dysregulation and the contribution of the adipose-derived signaling molecules to the progression of cardiomyopathy have been examined recently [54,55]. Recent findings from our laboratory also established the crucial role of adipocyte function in the pathophysiology of uremic cardiomyopathy [13]. It is of interest that many pathways associated with pathology in other organs (especially the heart) were noted in our analysis. Hence, the link between adipose tissues and systemic disorders evoking cardiac pathology seems strong.

Even though we are talking about a large number of molecular phenotypical alterations, it appears that cellular oxidant stress is a simple and profound root cause of many of these alterations. This is evidenced by the similarity of the in vivo changes to that produced in vitro as well as the success of targeting Na,K-ATPase oxidant amplification in both settings. Along these lines, while a plethora of abnormalities may be identified within the remarkable symptom complex of uremia, it appears that oxidant stress in general and, perhaps more importantly, such oxidant stress occurring within adipocytes may be a simple and effective target to focus therapeutic efforts on.

## 4. Materials and Methods

### 4.1. Experimental Design for In Vivo Studies

All animal studies were approved (IACUC No.: 11417053; Approval Date: 1 May 2019) by the Marshall University Animal Care Committee in accordance with the National Institutes of Health (NIH) Guidelines for Care and Use of Laboratory Animals. Male C57Bl6 mice (10–12 weeks old) were purchased from Hilltop Laboratory (Scottdale, PA, USA). Upon arrival to the Robert C. Byrd Biotechnology Science Center Animal Research Facility, mice were housed in a pathogen-free animal facility in designated rooms equipped with cages that supplied purified air under a 12-h light/dark cycle. Mice were fed a normal chow diet with ad libitum access to water. To mimic uremic cardiomyopathy, 5/6-nephrectomy (PNx) surgeries were performed on these mice as described previously [13]. Briefly, the PNx model uses a two-step surgical approach. The first step is to surgically ligate the superior and inferior poles of the left kidney so only one-third of the left kidney mass is functional. The second step is to remove the right kidney 7 days post-ligation. For sham controls, the two-step surgical procedure was repeated without ligation of the left kidney and removal of the right kidney.

Lentiviral vectors expressing either GFP-NaKtide or GFP cDNA under the control of an adiponectin promoter were constructed by VectorBuilder Inc (Chicago, IL, USA). to yield adipocyte-specific expression. Lentiviral constructs featured the adiponectin promoter driving expression of the NaKtide cassette linked by means of a 2A peptide to GFP for bicistronic expression. The 2A peptides are a class of 18–22 amino acid long self-cleaving peptide that maintains each protein as its own domain. Among the 2A peptides family, T2A was used for our lentiviral construct. Lentivirus (100 µL, 2 × 10^9^ TU/mL) with NaKtide or its counterpart Lenti-GFP in saline was intraperitoneally (IP) -injected into C57Bl6 mice as described previously [13], followed by PNx on the same day to experimentally induce uremic cardiomyopathy.

### 4.2. Experimental Design for In Vitro Studies

Frozen mouse pre-adipocytes (3T3-L1) were purchased from ATCC (Manassas, VA, USA) and re-suspended in Dulbecco’s Modified Eagle Media (DMEM). Mouse pre-adipocytes were then supplemented with 10% heat-inactivated fetal bovine serum and 1% antibiotic/antimycotic solution. The cells were plated at a density of 1–5 × 10^6^ cells per 25 cm^2^ dish and maintained at 37 °C in a 5% CO2 incubator. The medium was changed after 48 h and every 3–4 days thereafter. When the 3T3-L1 cells were confluent, the cells were recovered by the addition of trypsin [28]. The 3T3-L1 cells (passage 2–3) were plated in 6- and 24-well plates at a density of 10,000 cells/cm^2^ and cultured in DMEM to achieve at least 80% confluency. The medium was replaced with adipogenic medium and the cells were cultured for an additional five days [2]. Mouse adipocytes were treated with IS (100 μM) or oxLDL (50 μg/mL) with or without pNaKtide (0.7 μM), every 24 h for 2 days.

### 4.3. RNA-Seq and Data Analysis

Total RNA was extracted from adipose tissue using RNeasy Protect Mini Kit (QIAGEN, Germantown, MD, USA) as described previously [20]. The following in vivo groups were studied: (1) Sham + adipo-GFP (Control), (2) sham + adipo-NaKtide (NaKtide), (3) PNx + adipo-GFP (PNx), and (4) PNx + adipo-NaKtide. Six replicates were studied in these 4 in vivo groups. The following in vitro groups were studied: (1) 3T3L1 preadipocytes treated with adipogenic media alone (Control), (2) pNaKtide alone, (3) IS, (4) IS + pNaKtide, (5) oxLDL and (6) oxLDL + pNaKtide. Five replicates were studied in these in vitro groups.

All samples had RNA integrity numbers greater than 8. Libraries were prepared from the total RNA per sample and library quality was assessed by electrophoretic analysis on the Agilent BioAnalyzer 2100 system. RNA-Seq libraries were sequenced in a 2 × 50 paired-end design on an Illumina HiSeq 1500. A complete RNA-Seq analysis was performed by Marshall University Genomic Core. Reads were trimmed to remove adapter sequence and low-quality base calls using Trimmomatic v0.38 and aligned to the reference mouse genome GRCm38 using HISAT2 v2.1.0. A count table of reads per gene per sample was created using the R/Bioconductor package Genomic Alignments v1.16.0 [56]. Differential gene expression was established with a false discovery rate (FDR) threshold of 0.1 using the DESeq2 package [57]. Genes were examined for overrepresentation analysis (ORA) using the Reactome dataset employing the ReactomePA [58] and clusterProfiler [59] R packages. Additional data sets were queried using the WebGestalt toolkit including “KEGG” pathways (www.webgestalt.org) [60]. Gene sequence enrichment analysis (GSEA) was also performed using the fgsea [61] and gage [62] R packages. The KEGG pathways were specifically queried with a gmt file (c2.cp.kegg.v7.1entrez.gmt) obtained at the KEGG website https://www.genome.jp/kegg/pathway.html. Results obtained with the R routine and those obtained with the WebGestalt toolkit were essentially identical when the same database was queried. Selected KEGG pathway analyses were displayed either with heat maps constructed with fpkm data or utilizing the pathview [63] R package.

Topological analysis was performed using the WGCNA R package [64]. First, the in vivo gene expression was examined in concert with in vivo characteristics which we have previously reported [13]. Evaluation of initial groupings did not show a need to exclude any in vivo or in vitro group. We next established a soft threshold for network construction by examining topological characteristics over a range from 2 to 40. After analysis of both the in vivo and in vitro data sets, it was decided to choose a power of 20 for all further calculations (Figure 2B). Subsets of genes were determined by merging those that had cut differences of <0.01 which allowed for dendrogram and module construction, and for the in vivo data, correlation of key in vivo parameters with the specific modules. For the in vitro data, we compared the effects of IS and oxLDL by separating the in vitro data into 2 sets of 4 groups. Both sets contained the control and pNaKtide groups; one set included the IS and IS + pNaKtide groups while the other included oxLDL and oxLDL + pNaKtide. Once these comparisons were made, we then compared the in vivo gene expressions with the in vitro gene expressions, merging the IS and oxLDL sets. Topological characteristics were compared using the aforementioned WGCNA package [65], following the step by step instructions at the website https://horvath.genetics.ucla.edu/html/CoexpressionNetwork/Rpackages/WGCNA/Tutorials/.

Raw sequencing reads have been deposited at the Gene Expression Omnibus at the National Center for Biotechnology Information and can be accessed using the accession number GSE152725.

### 4.4. Real-Time Reverse Transcriptase Polymerase Chain Reaction (qRT-PCR) for RNASeq Validation

In order to validate the high throughput RNA-sequencing data, previously extracted total RNA from visceral adipose tissue of our in vivo groups were used to perform qRT-PCR reactions. Briefly, following total RNA extraction and preparation of cDNA libraries, qRT-PCR reactions were performed in triplicate using SYBR Green PCR Master Mix on a 7500 HT Fast Real-Time PCR System (Applied Biosystems, Foster City, CA, USA), as described previously [13]. The relative mRNA expression levels of eight differentially expressed genes, related to oxidative stress and inflammation, was determined. These genes included AKT (gene symbol: *Akt3*), ERK (gene symbol: *Mapk1*), PKC (gene Symbol: *Prkca*), eNOS (gene symbol: *Nos3*), IKK (gene symbol: *Chuk*), TLR4 (gene Symbol: *Tlr4*), CDK (gene symbol: *Cdkn1a*) and CREB (gene symbol: *Creb*). The fold changes were calculated from relative expression for PNx versus Control and PNx + NaKtide versus PNx, followed by estimation of log_2_ Fold Change values per candidate gene to compare RNAseq and qRT-PCR findings.

### 4.5. Statistical Analyses

Data from RNA-Seq experiments are presented in “volcano” plots as the anti-log10 of the raw *p*-value against the base 2 log of the fold change (“log_2_ fold change”; Figure 1A, Figure 4A and Figure 5A). A Benjamini–Hochberg adjusted *p*-value of 0.1 was used as a threshold for the ORA analysis [57]. In order to keep naming consistent throughout, all raw *p*-values were called “unadjusted *p*-value” and all adjusted *p*-values are called “BH-adjusted *p*-value”. For the GSEA, the entire gene dataset was used allowing the GSEA algorithm to apply the log_2_ fold change data to determine whether gene sets were coordinately over or under expressed [62]. The significance values for the overrepresentation of different pathways were calculated with the fgsea R package. Virtually identical gene pathways with similar estimated *p*-values were identified by querying the WebGestalt website with the overall set of genes and log_2_ fold change values observed.

In the case of topological analysis and, in particular, the comparison of different gene expression sets, we focused on highly correlated gene expressions along with (in the case of the in vivo data set) phenotypical features using the WGCNA R package as has been reported recently [65].

## Supplementary Materials

The following are available online at https://www.mdpi.com/1422-0067/21/16/5923/s1. Table S1: Summary of enriched KEGG pathways under experimental renal failure in PNx mice. Table S2: Summary of enriched KEGG pathways in 3T3L1 adipocytes treated with oxLDL. Table S3: Summary of enriched KEGG pathways in 3T3L1 adipocytes treated with IS. Figure S1: Functional enrichment analyses using Kyoto Encyclopedia of Genes and Genomes (KEGG) pathways in PNx mice model. Figure S2: KEGG pathway analysis in adipose tissue of PNx mice model with alteration of key genes associated with uremic cardiomyopathy. Figure S3: In vivo phenotypical features characterized with ORA and the associated heat maps of GSEA scores. Figure S4: Functional enrichment analyses using Kyoto Encyclopedia of Genes and Genomes (KEGG) pathways in oxLDL treated murine adipocytes with alteration of key genes associated with oxidative stress. Figure S5: Functional enrichment analyses using Kyoto Encyclopedia of Genes and Genomes (KEGG) pathways in IS treated murine adipocytes with alteration of key genes associated with oxidative stress. Figure S6: Heat maps of gene expression associated with enriched KEGG pathways by OxLDL and IS using GSEA. Figure S7: Network diagram associated with the analysis of genes in STRING database and the heat maps of gene expression associated KEGG pathways altered in vitro. Figure S8: Scale independence and mean connectivity as a function of soft threshold in in vitro datasets.
