# Supplementary material for: Oxidant-Induced Alterations in the Adipocyte Transcriptome: Role of the Na,K-ATPase Oxidant Amplification Loop
Source: Int J Mol Sci. 2020 Aug 18;21(16):5923. doi: 10.3390/ijms21165923 (PMC7460641; doi:10.3390/ijms21165923)
Supplement: Supplementary file 1 [file ijms-21-05923-s001.pdf]

Supplementary Materials for

Oxidant-Induced Alterations in the Adipocyte Transcriptome: Role of the Na,K-ATPase Oxidant Amplification Loop

Komal Sodhi[1], James Denvir[1], Jiang Liu[1], Juan R. Sanabria[1], Yiliang Chen[2], Roy Silverstein[2], Zijian Xie[1†], Nader G. Abraham[3], and Joseph I. Shapiro[1*]

Correspondence to: shapiroj@marshall.edu

Supplementary Material Table of Contents

Table S1: Summary of enriched KEGG pathways under experimental renal failure in PNx mice.

Table S2: Summary of enriched KEGG pathways in 3T3L1 adipocytes treated with oxLDL.

Table S3: Summary of enriched KEGG pathways in 3T3L1 adipocytes treated with IS.

Figure S1: Functional enrichment analyses using Kyoto Encyclopedia of Genes and Genomes (KEGG) pathways in PNx mice model.

Figure S2: KEGG pathway analysis in adipose tissue of PNx mice model with alteration of key genes associated with uremic cardiomyopathy.

Figure S3: *In vivo* phenotypical features characterized with ORA and the associated heat maps of GSEA scores.

Figure S4: Functional enrichment analyses using Kyoto Encyclopedia of Genes and Genomes (KEGG) pathways in oxLDL treated murine adipocytes with alteration of key genes associated with oxidative stress.

Figure S5: Functional enrichment analyses using Kyoto Encyclopedia of Genes and Genomes (KEGG) pathways in IS treated murine adipocytes with alteration of key genes associated with oxidative stress.

Figure S6: Heat maps of gene expression associated with enriched KEGG pathways by OxLDL and IS using GSEA.

Figure S7: Network diagram associated with the analysis of genes in STRING database and the heatmaps of gene expression associated KEGG pathways altered *in vitro*.

Figure S8: Scale independence and Mean connectivity as a function of Soft Threshold in in vitro datasets.

KEGG Pathway	-log10 (p value)
GALACTOSE METABOLISM	2.9
ASCORBATE AND ALDARATE METABOLISM	2.9
ALANINE ASPARTATE AND GLUTAMATE METABOLISM	2.9
GLYCINE SERINE AND THREONINE METABOLISM	2.9
CYSTEINE AND METHIONINE METABOLISM	2.9
LYSINE DEGRADATION	2.9
HISTIDINE METABOLISM	2.9
PHENYLALANINE METABOLISM	2.9
BETA ALANINE METABOLISM	2.9
TAURINE AND HYPOTAURINE METABOLISM	2.9
LINOLEIC ACID METABOLISM	2.9
PROPANOATE METABOLISM	2.9
BUTANOATE METABOLISM	2.9
FOLATE BIOSYNTHESIS	2.9
LIMONENE AND PINENE DEGRADATION	2.9
NITROGEN METABOLISM	2.9
RENIN ANGIOTENSIN SYSTEM	2.9
TASTE TRANSDUCTION	2.9
MATURITY ONSET DIABETES OF THE YOUNG	2.9
PROXIMAL TUBULE BICARBONATE RECLAMATION	2.9
CITRATE CYCLE TCA CYCLE	2.8
PENTOSE PHOSPHATE PATHWAY	2.8
TYROSINE METABOLISM	2.8
TRYPTOPHAN METABOLISM	2.8
GLYCEROLIPID METABOLISM	2.8
PYRUVATE METABOLISM	2.8
RIBOFLAVIN METABOLISM	2.8
NICOTINATE AND NICOTINAMIDE METABOLISM	2.8
PORPHYRIN AND CHLOROPHYLL METABOLISM	2.8
DRUG METABOLISM OTHER ENZYMES	2.8
ABC TRANSPORTERS	2.8
VASOPRESSIN REGULATED WATER REABSORPTION	2.8
ASTHMA	2.8
PRIMARY IMMUNODEFICIENCY	2.8
PRION DISEASES	2.7
FRUCTOSE AND MANNOSE METABOLISM	2.6
FATTY ACID METABOLISM	2.6
STEROID HORMONE BIOSYNTHESIS	2.6
VALINE LEUCINE AND ISOLEUCINE DEGRADATION	2.6
ARGININE AND PROLINE METABOLISM	2.6

GLUTATHIONE METABOLISM	2.6
STARCH AND SUCROSE METABOLISM	2.6
INOSITOL PHOSPHATE METABOLISM	2.6
ARACHIDONIC ACID METABOLISM	2.6
RETINOL METABOLISM	2.6
PROTEASOME	2.6
NUCLEOTIDE EXCISION REPAIR	2.6
MTOR SIGNALING PATHWAY	2.6
HEDGEHOG SIGNALING PATHWAY	2.6
INTESTINAL IMMUNE NETWORK FOR IGA PRODUCTION	2.6
OLFACTORY TRANSDUCTION	2.6
TYPE I DIABETES MELLITUS	2.6
ALDOSTERONE REGULATED SODIUM REABSORPTION	2.6
VIBRIO CHOLERAE INFECTION	2.6
BLADDER CANCER	2.6
ALLOGRAFT REJECTION	2.6
GRAFT VERSUS HOST DISEASE	2.6
TYPE II DIABETES MELLITUS	2.5
BASAL CELL CARCINOMA	2.4
AMINO SUGAR AND NUCLEOTIDE SUGAR METABOLISM	2.3
ETHER LIPID METABOLISM	2.2
AMYOTROPHIC LATERAL SCLEROSIS ALS	2.2
ENDOMETRIAL CANCER	2.2
OTHER GLYCAN DEGRADATION	2.1
NOD LIKE RECEPTOR SIGNALING PATHWAY	2.1
GLYCOLYSIS GLUCONEOGENESIS	2
METABOLISM OF XENOBIOTICS BY CYTOCHROME P450	2
P53 SIGNALING PATHWAY	2
EPITHELIAL CELL SIGNALING IN HELICOBACTER PYLORI INFECTION	2
PATHOGENIC ESCHERICHIA COLI INFECTION	2
ACUTE MYELOID LEUKEMIA	2
NON SMALL CELL LUNG CANCER	2
AUTOIMMUNE THYROID DISEASE	2
GLYCEROPHOSPHOLIPID METABOLISM	1.9
THYROID CANCER	1.8
GLYOXYLATE AND DICARBOXYLATE METABOLISM	1.7
DRUG METABOLISM CYTOCHROME P450	1.7
PEROXISOME	1.7
RIG I LIKE RECEPTOR SIGNALING PATHWAY	1.7
COLORECTAL CANCER	1.7
GLIOMA	1.6

VALINE LEUCINE AND ISOLEUCINE BIOSYNTHESIS	1.5
ADIPOCYTOKINE SIGNALING PATHWAY	1.4
PPAR SIGNALING PATHWAY	1.3
PHOSPHATIDYLINOSITOL SIGNALING SYSTEM	1.3
CIRCADIAN RHYTHM MAMMAL	1.3
LONG TERM DEPRESSION	1.3
NOTCH SIGNALING PATHWAY	1.2
PYRIMIDINE METABOLISM	1.1
PANTOTHENATE AND COA BIOSYNTHESIS	1.1
BASE EXCISION REPAIR	1.1
CARDIAC MUSCLE CONTRACTION	1.1
VEGF SIGNALING PATHWAY	1.1
ADHERENS JUNCTION	1.1
LONG TERM POTENTIATION	1.1
RENAL CELL CARCINOMA	1.1
MELANOMA	1.1
ARRHYTHMOGENIC RIGHT VENTRICULAR CARDIOMYOPATHY ARVC	1.1
VIRAL MYOCARDITIS	1.1
CYTOSOLIC DNA SENSING PATHWAY	1

Table S1: Summary of enriched KEGG pathways under experimental renal failure in PNx mice. Datasets were queried using the WebGestalt toolkit including “KEGG” pathways and GSEA analysis was performed using R packages. GSEA analysis showed 114 dysregulated KEGG pathways under experimental renal failure *in vivo*.

KEGG Pathways	-log10(p value)
GLYCOLYSIS GLUCONEOGENESIS	5.2
FATTY ACID METABOLISM	5.2
OXIDATIVE PHOSPHORYLATION	5.2
GLYCINE SERINE AND THREONINE METABOLISM	5.2
VALINE LEUCINE AND ISOLEUCINE DEGRADATION	5.2
ARGININE AND PROLINE METABOLISM	5.2
TYROSINE METABOLISM	5.2
BETA ALANINE METABOLISM	5.2
GLUTATHIONE METABOLISM	5.2
PYRUVATE METABOLISM	5.2
BUTANOATE METABOLISM	5.2
METABOLISM OF XENOBIOTICS BY CYTOCHROME P450	5.2
DRUG METABOLISM CYTOCHROME P450	5.2
PROTEASOME	5.2
ERBB SIGNALING PATHWAY	5.2
LYSOSOME	5.2
VASCULAR SMOOTH MUSCLE CONTRACTION	5.2
ECM RECEPTOR INTERACTION	5.2
CELL ADHESION MOLECULES CAMS	5.2
COMPLEMENT AND COAGULATION CASCADES	5.2
ANTIGEN PROCESSING AND PRESENTATION	5.2
RENIN ANGIOTENSIN SYSTEM	5.2
HEMATOPOIETIC CELL LINEAGE	5.2
LONG TERM POTENTIATION	5.2
GNRH SIGNALING PATHWAY	5.2
PROGESTERONE MEDIATED OOCYTE MATURATION	5.2
TYPE I DIABETES MELLITUS	5.2
PRION DISEASES	5.2
COLORECTAL CANCER	5.2
GLIOMA	5.2
PROSTATE CANCER	5.2
MELANOMA	5.2
AUTOIMMUNE THYROID DISEASE	5.2
ALLOGRAFT REJECTION	5.2
GRAFT VERSUS HOST DISEASE	5.2
ARRHYTHMOGENIC RIGHT VENTRICULAR CARDIOMYOPATHY ARVC	5.2
VIRAL MYOCARDITIS	5.2
ALZHEIMERS DISEASE	5.1
HUNTINGTONS DISEASE	5.1
CALCIUM SIGNALING PATHWAY	5

CHEMOKINE SIGNALING PATHWAY	4.9
FOCAL ADHESION	4.9
CYTOKINE CYTOKINE RECEPTOR INTERACTION	4.8
NEUROACTIVE LIGAND RECEPTOR INTERACTION	4.8
TGF BETA SIGNALING PATHWAY	4.8
GAP JUNCTION	4.8
LEISHMANIA INFECTION	4.8
CHRONIC MYELOID LEUKEMIA	4.8
SMALL CELL LUNG CANCER	4.8
DILATED CARDIOMYOPATHY	4.8
PROPANOATE METABOLISM	4.7
SPLICEOSOME	4.7
TASTE TRANSDUCTION	4.7
PATHWAYS IN CANCER	4.6
P53 SIGNALING PATHWAY	4.5
CARDIAC MUSCLE CONTRACTION	4.4
RIBOSOME	4.3
SNARE INTERACTIONS IN VESICULAR TRANSPORT	4.2
NITROGEN METABOLISM	4.1
JAK STAT SIGNALING PATHWAY	4
VIBRIO CHOLERAE INFECTION	3.9
PANCREATIC CANCER	3.8
TRYPTOPHAN METABOLISM	3.7
MAPK SIGNALING PATHWAY	3.7
CELL CYCLE	3.7
OLFACTORY TRANSDUCTION	3.7
PARKINSONS DISEASE	3.7
NON SMALL CELL LUNG CANCER	3.6
HISTIDINE METABOLISM	3.5
ALDOSTERONE REGULATED SODIUM REABSORPTION	3.4
PROXIMAL TUBULE BICARBONATE RECLAMATION	3.3
EPITHELIAL CELL SIGNALING IN HELICOBACTER PYLORI INFECTION	3.3
ASTHMA	3.3
PRIMARY IMMUNODEFICIENCY	3.3
ARACHIDONIC ACID METABOLISM	3.2
NATURAL KILLER CELL MEDIATED CYTOTOXICITY	3.1
AMYOTROPHIC LATERAL SCLEROSIS ALS	3
MELANOGENESIS	2.9
MATURITY ONSET DIABETES OF THE YOUNG	2.8
BLADDER CANCER	2.7
CITRATE CYCLE TCA CYCLE	2.6

RETINOL METABOLISM	2.5
PURINE METABOLISM	2.4
LIMONENE AND PINENE DEGRADATION	2.3
PPAR SIGNALING PATHWAY	2.2
ENDOMETRIAL CANCER	2.1
ADHERENS JUNCTION	2
TAURINE AND HYPOTAURINE METABOLISM	1.9
ENDOCYTOSIS	1.8
PORPHYRIN AND CHLOROPHYLL METABOLISM	1.7
GALACTOSE METABOLISM	1.6
VALINE LEUCINE AND ISOLEUCINE BIOSYNTHESIS	1.5
NUCLEOTIDE EXCISION REPAIR	1.4
HYPERTROPHIC CARDIOMYOPATHY HCM	1.3
OOCYTE MEIOSIS	1.2
ALANINE ASPARTATE AND GLUTAMATE METABOLISM	1.1
VASOPRESSIN REGULATED WATER REABSORPTION	1.0

Table S2: Summary of enriched KEGG pathways in 3T3L1 adipocytes treated with oxLDL. Datasets were queried using the WebGestalt toolkit including “KEGG” pathways and GSEA analysis was performed using R packages. GSEA analysis showed 107 dysregulated KEGG pathways oxLDL treated 3T3L1 adipocytes *in vitro*.

KEGG Pathways	-log10(p value)
GLYCOLYSIS_GLUCONEOGENESIS	2.4
FATTY_ACID_METABOLISM	2.4
VALINE_LEUCINE_AND_ISOLEUCINE_DEGRADATION	2.4
ARGININE_AND_PROLINE_METABOLISM	2.4
PYRUVATE_METABOLISM	2.4
METABOLISM_OF_XENOBIOTICS_BY_CYTOCHROME_P450	2.4
DRUG_METABOLISM_CYTOCHROME_P450	2.4
SPLICEOSOME	2.4
ERBB_SIGNALING_PATHWAY	2.4
VASCULAR_SMOOTH_MUSCLE_CONTRACTION	2.4
ECM_RECEPTOR_INTERACTION	2.4
COMPLEMENT_AND_COAGULATION_CASCADES	2.4
ANTIGEN_PROCESSING_AND_PRESENTATION	2.4
HEMATOPOIETIC_CELL_LINEAGE	2.4
GNRH_SIGNALING_PATHWAY	2.4
PROGESTERONE_MEDIATED_OOCYTE_MATURATION	2.4
GLIOMA	2.4
PROSTATE_CANCER	2.4
SMALL_CELL_LUNG_CANCER	2.4
VIRAL_MYOCARDITIS	2.4
BUTANOATE_METABOLISM	2.4
CALCIUM_SIGNALING_PATHWAY	2.4
CHEMOKINE_SIGNALING_PATHWAY	2.4
FOCAL_ADHESION	2.4
TYPE_I_DIABETES_MELLITUS	2.4
ALZHEIMERS_DISEASE	2.4
ALLOGRAFT_REJECTION	2.4
GRAFT_VERSUS_HOST_DISEASE	2.4
BETA_ALANINE_METABOLISM	2.4
NEUROACTIVE_LIGAND_RECEPTOR_INTERACTION	2.4
GAP_JUNCTION	2.4
LONG_TERM_POTENTIATION	2.4
TASTE_TRANSDUCTION	2.4
MELANOMA	2.4
ARRHYTHMOGENIC_RIGHT_VENTRICULAR_CARDIOMYOPATHY_ARVC	2.4
TYROSINE_METABOLISM	2.3
PATHWAYS_IN_CANCER	2.3
AUTOIMMUNE_THYROID_DISEASE	2.3
HUNTINGTONS_DISEASE	2.3
OXIDATIVE_PHOSPHORYLATION	2.2

CYTOKINE_CYTOKINE_RECEPTOR_INTERACTION	2.2
MELANOGENESIS	2.1
DILATED_CARDIOMYOPATHY	2.1
EPITHELIAL_CELL_SIGNALING_IN_HELICOBACTER_PYLORI_INFECTION	2.1
NITROGEN_METABOLISM	2.1
SNARE_INTERACTIONS_IN_VESICULAR_TRANSPORT	2.1
LEUKOCYTE_TRANSENDOTHELIAL_MIGRATION	2.1
CELL_ADHESION_MOLECULES_CAMS	2.1
COLORECTAL_CANCER	2.0
OLFACTORY_TRANSDUCTION	2.0
PRION_DISEASES	1.9
GLUTATHIONE_METABOLISM	1.9
ENDOMETRIAL_CANCER	1.9
NON_SMALL_CELL_LUNG_CANCER	1.9
NUCLEOTIDE_EXCISION_REPAIR	1.9
NEUROTROPHIN_SIGNALING_PATHWAY	1.9
PRIMARY_IMMUNODEFICIENCY	1.9
PROPANOATE_METABOLISM	1.9
OOCYTE_MEIOSIS	1.8
LONG_TERM_DEPRESSION	1.8
VALINE_LEUCINE_AND_ISOLEUCINE_BIOSYNTHESIS	1.7
JAK_STAT_SIGNALING_PATHWAY	1.7
PPAR_SIGNALING_PATHWAY	1.7
AMYOTROPHIC_LATERAL_SCLEROSIS_ALS	1.7
GLYCINE_SERINE_AND_THREONINE_METABOLISM	1.7
LYSOSOME	1.7
TGF_BETA_SIGNALING_PATHWAY	1.7
MATURITY_ONSET_DIABETES_OF_THE_YOUNG	1.7
PROXIMAL_TUBULE_BICARBONATE_RECLAMATION	1.7
ALDOSTERONE_REGULATED_SODIUM_REABSORPTION	1.6
LIMONENE_AND_PINENE_DEGRADATION	1.6
ASTHMA	1.6
HISTIDINE_METABOLISM	1.6
ALANINE_ASPARTATE_AND_GLUTAMATE_METABOLISM	1.6
PANCREATIC_CANCER	1.6
CHRONIC_MYELOID_LEUKEMIA	1.5
VIBRIO_CHOLERAE_INFECTION	1.5
PURINE_METABOLISM	1.5
OTHER_GLYCAN_DEGRADATION	1.5
MISMATCH_REPAIR	1.5
ARACHIDONIC_ACID_METABOLISM	1.4

REGULATION_OF_ACTIN_CYTOSKELETON	1.4
ADHERENS_JUNCTION	1.4
PANTOTHENATE_AND_COA_BIOSYNTHESIS	1.4
NATURAL_KILLER_CELL_MEDIATED_CYTOTOXICITY	1.4
TRYPTOPHAN_METABOLISM	1.3
PORPHYRIN_AND_CHLOROPHYLL_METABOLISM	1.3
RENIN_ANGIOTENSIN_SYSTEM	1.3
INSULIN_SIGNALING_PATHWAY	1.3
LEISHMANIA_INFECTION	1.3
T_CELL_RECEPTOR_SIGNALING_PATHWAY	1.3
SULFUR_METABOLISM	1.3
BLADDER_CANCER	1.3
HEDGEHOG_SIGNALING_PATHWAY	1.2
LINOLEIC_ACID_METABOLISM	1.2
TAURINE_AND_HYPOTAURINE_METABOLISM	1.2
GLYCOSAMINOGLYCAN_BIOSYNTHESIS_HEPARAN_SULFATE	1.2
CARDIAC_MUSCLE_CONTRACTION	1.2
TYPE_II_DIABETES_MELLITUS	1.1
GALACTOSE_METABOLISM	1.1
GLYCOSAMINOGLYCAN_BIOSYNTHESIS_CHONDROITIN_SULFATE	1.1
DNA_REPLICATION	1.1
B_CELL_RECEPTOR_SIGNALING_PATHWAY	1.1
VASOPRESSIN_REGULATED_WATER_REABSORPTION	1.1
PARKINSONS_DISEASE	1.1
VEGF_SIGNALING_PATHWAY	1.1
HYPERTROPHIC_CARDIOMYOPATHY_HCM	1.0

Table S3: Summary of enriched KEGG pathways in 3T3L1 adipocytes treated with IS. Datasets were queried using the WebGestalt toolkit including “KEGG” pathways and GSEA analysis was performed using R packages. GSEA analysis showed 107 dysregulated KEGG pathways in IS treated 3T3L1 adipocytes *in vitro*.

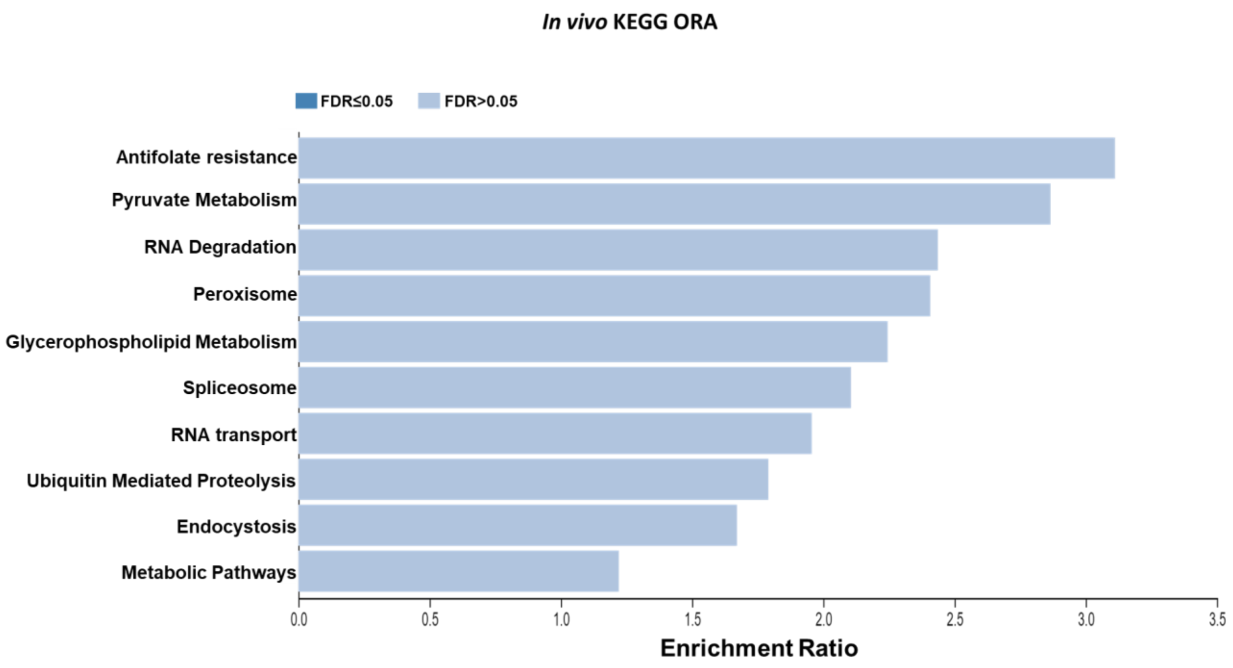

Supplementary Figure 1: Functional enrichment analyses using Kyoto Encyclopedia of Genes and Genomes (KEGG) pathways in PNx mice model. Over expression analysis of KEGG pathways, performed on the entire data set, with the highest differentially expressed genes.

A

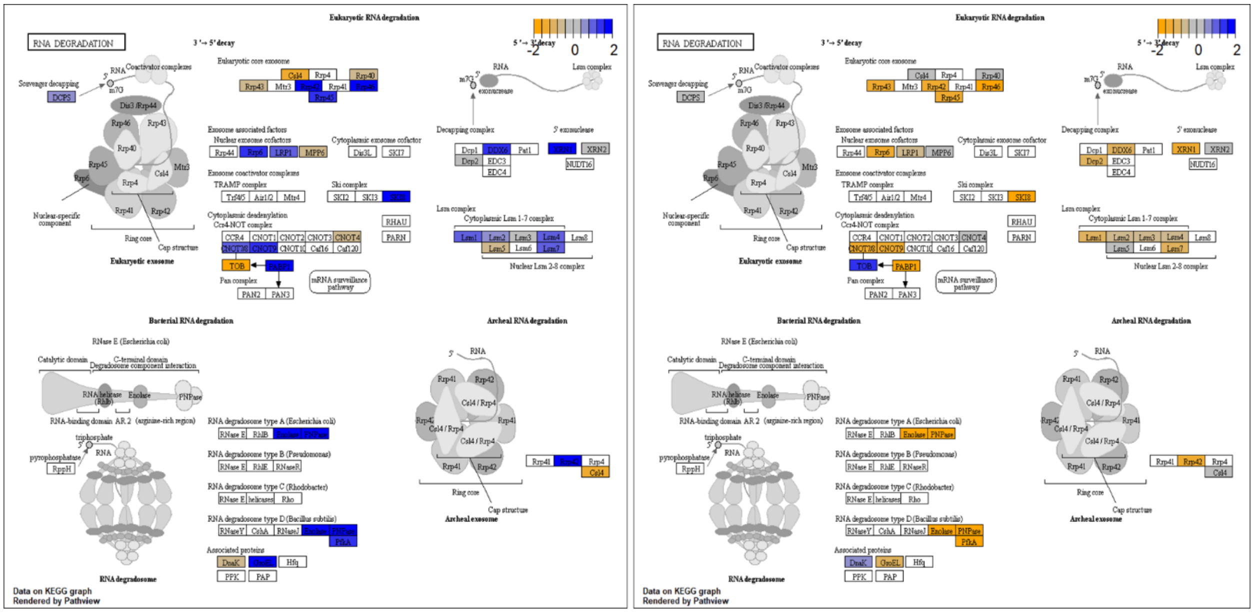

PNx PNx+NaKtide

B

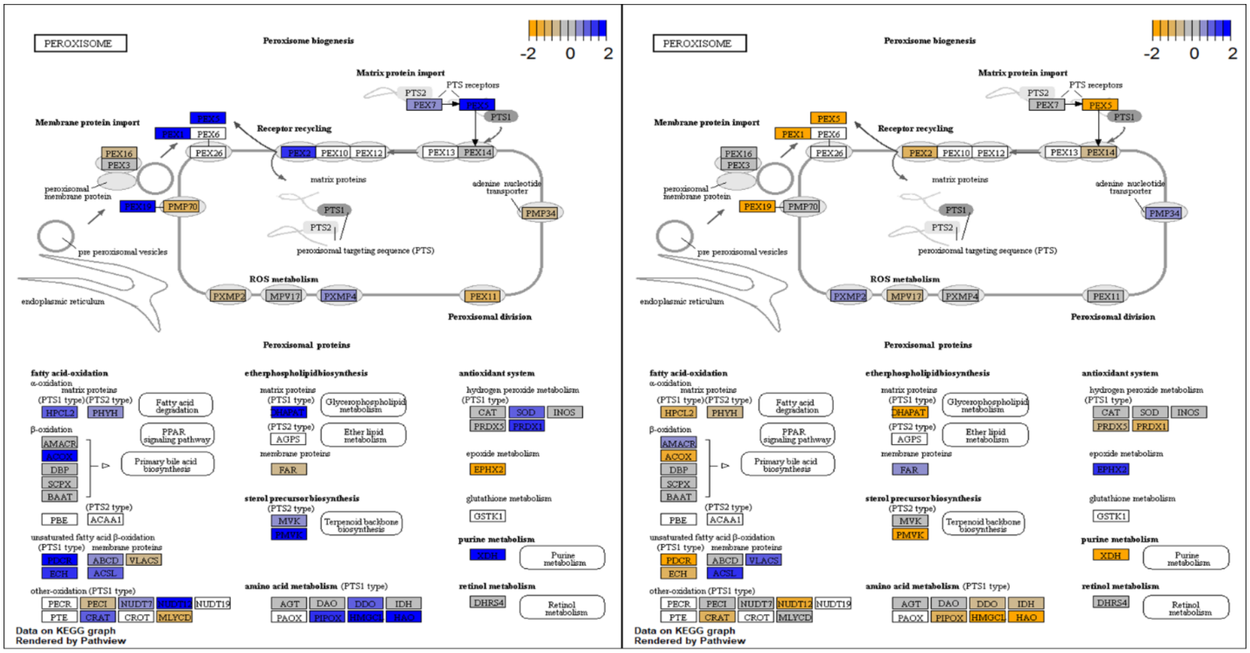

PNx PNx+NaKtide

C

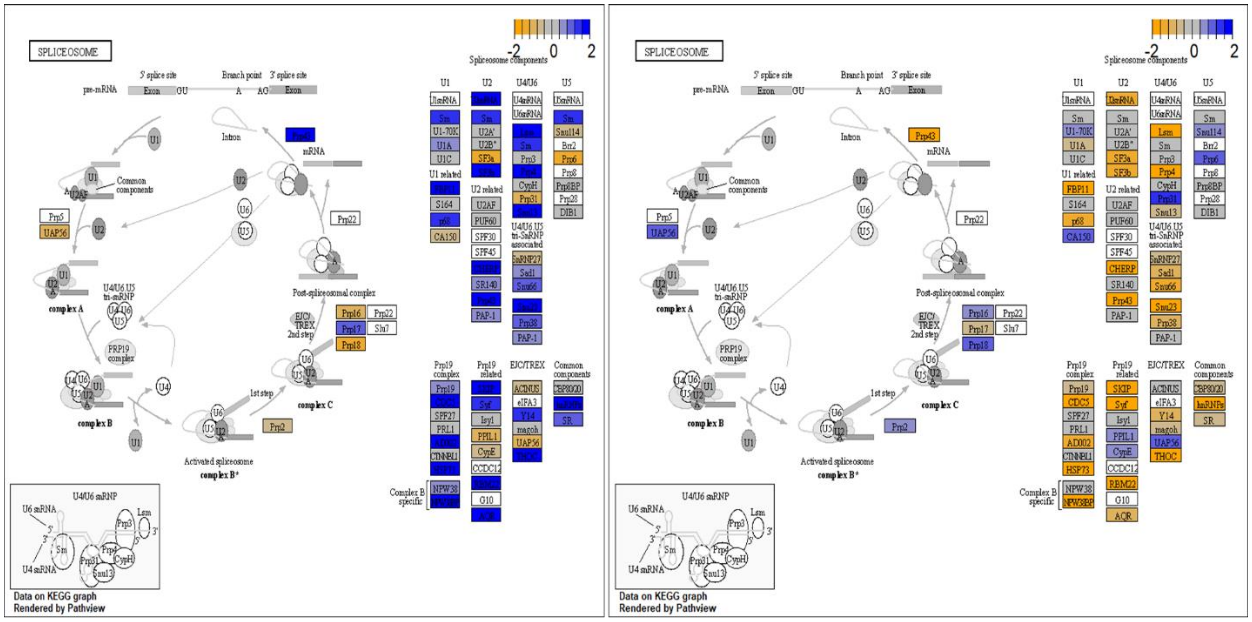

PNx PNx+NaKtide

D

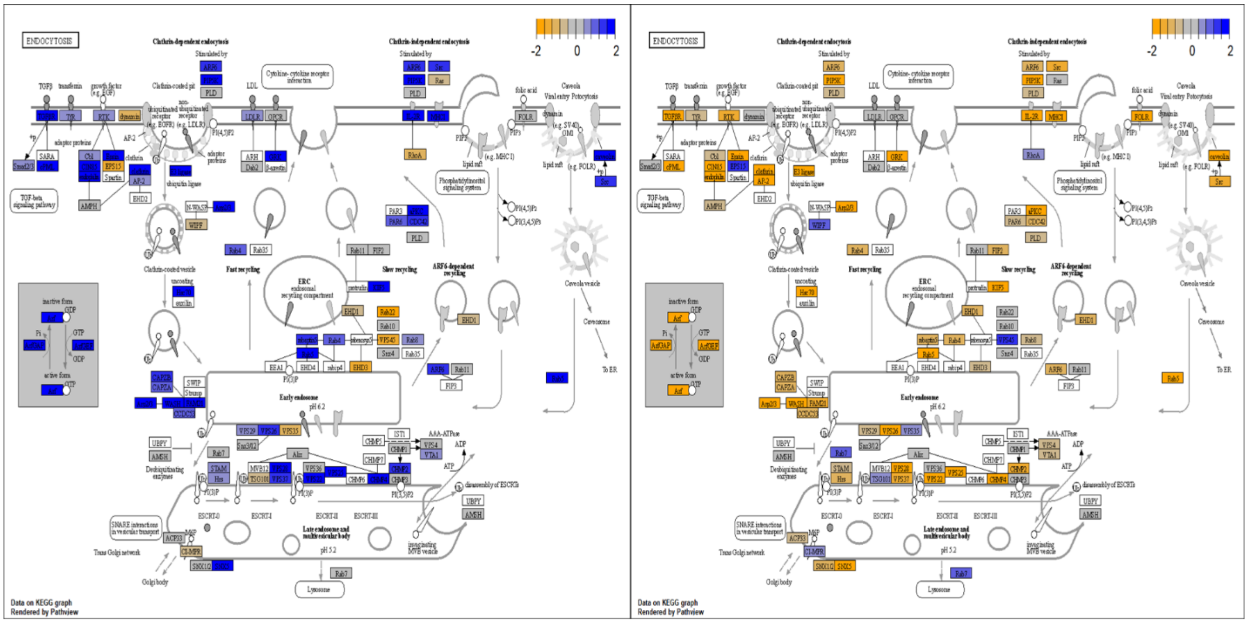

PNx PNx+NaKtide

Supplementary Figure 2: KEGG pathway analysis in adipose tissue of PNx mice model with alteration of key genes associated with uremic cardiomyopathy. Genes upregulated or downregulated in pathways associated with (A) RNA Degradation, (B) Peroxisome, (C) Spliceosome and (D) Endocytosis. Blue represent genes upregulated and orange represent genes downregulated.

GSEA Analysis and Heat Map Data from Light Green Module

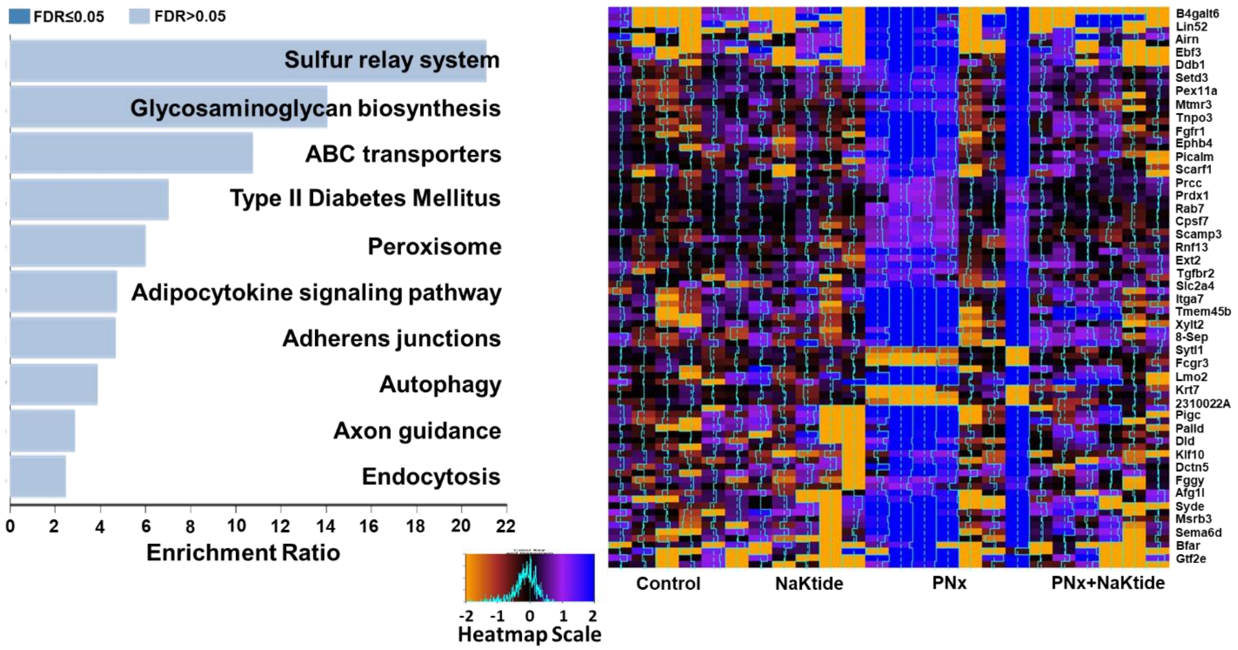

Supplemental Figure 3A: The *in vivo* KEGG ORA and the associated heat maps of GSEA scores: The figure shows the *in vivo* KEGG ORA (left panel) and the associated heat maps of GSEA scores (right panel) for groups of gene expression corresponding to the specific trait, cardiac fibrosis. The data shows significant dysregulation of genes associated with the trait under PNx condition and the possible reversal by NaKtide treatment.

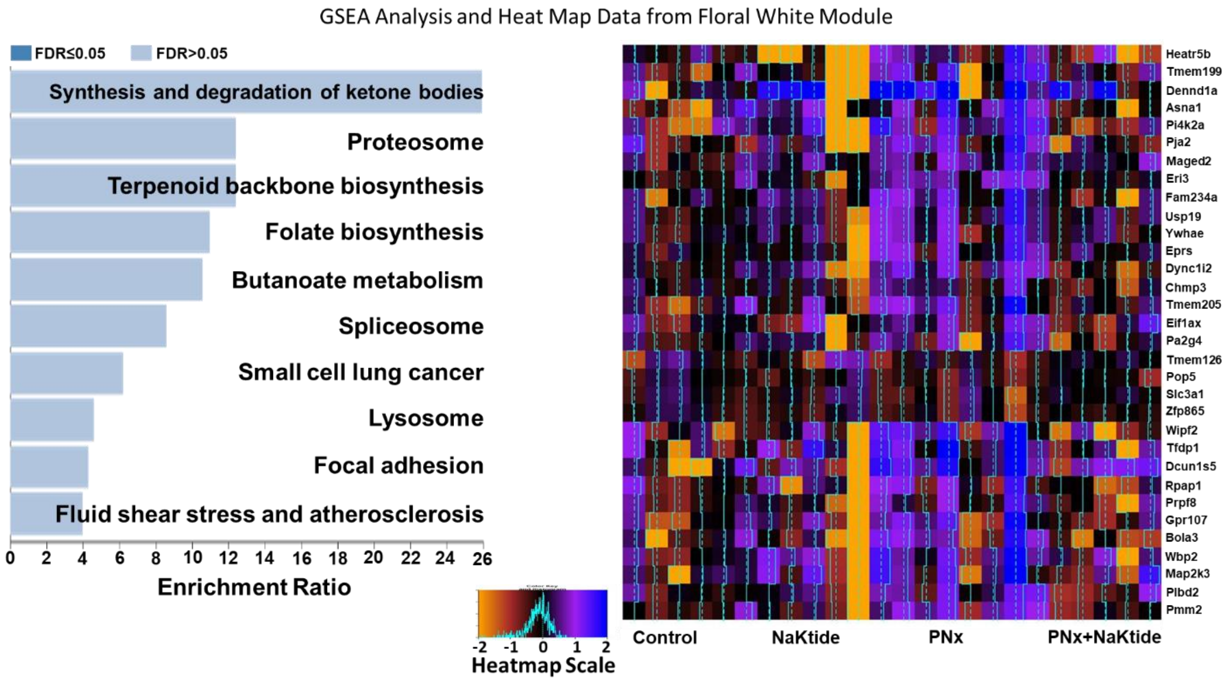

Supplemental Figure 3B: The *in vivo* KEGG ORA and the associated heat maps of GSEA scores. The figure shows the *in vivo* KEGG ORA (left panel) and the associated heat maps of GSEA scores (right panel) for groups of gene expression corresponding to the specific trait, left ventricular mass. The data shows significant dysregulation of genes associated with the trait under PNx condition and the possible reversal by NaKtide treatment.

GSEA Analysis and Heat Map Data from Sky Blue 3 Module

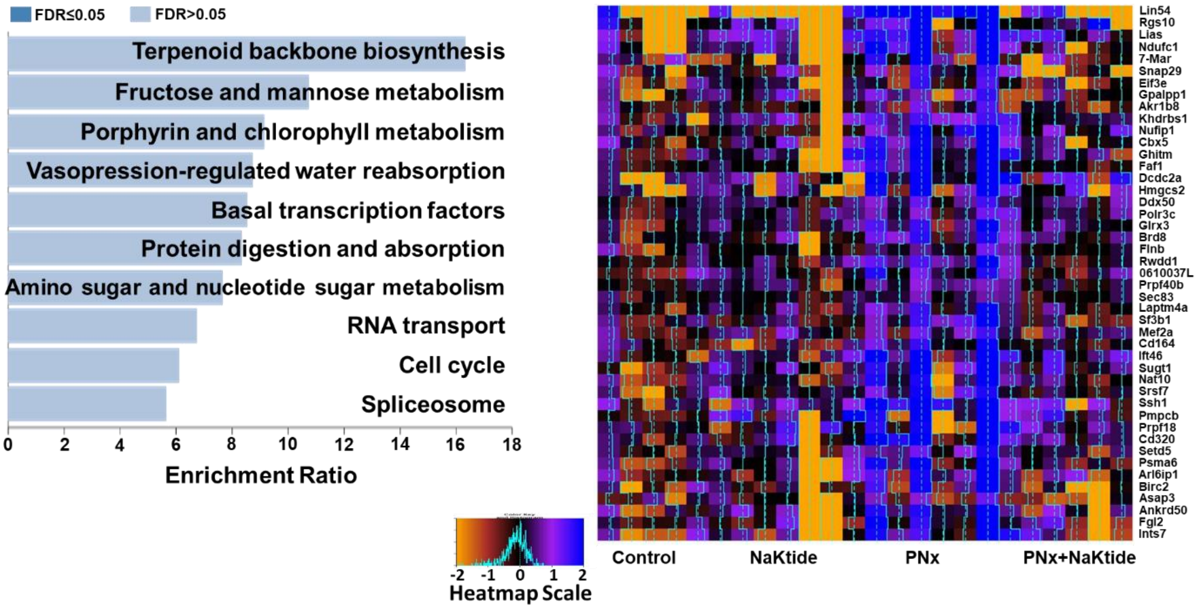

Supplemental Figure 3C: The *in vivo* KEGG ORA and the associated heat maps of GSEA scores. The figure shows the *in vivo* KEGG ORA (left panel) and the associated heat maps of GSEA scores (right panel) for groups of gene expression corresponding to the specific trait, MPI. The data shows significant dysregulation of genes associated with MPI under PNx condition and the possible reversal by NaKtide treatment.

GSEA Analysis and Heat Map Data from Plum Module

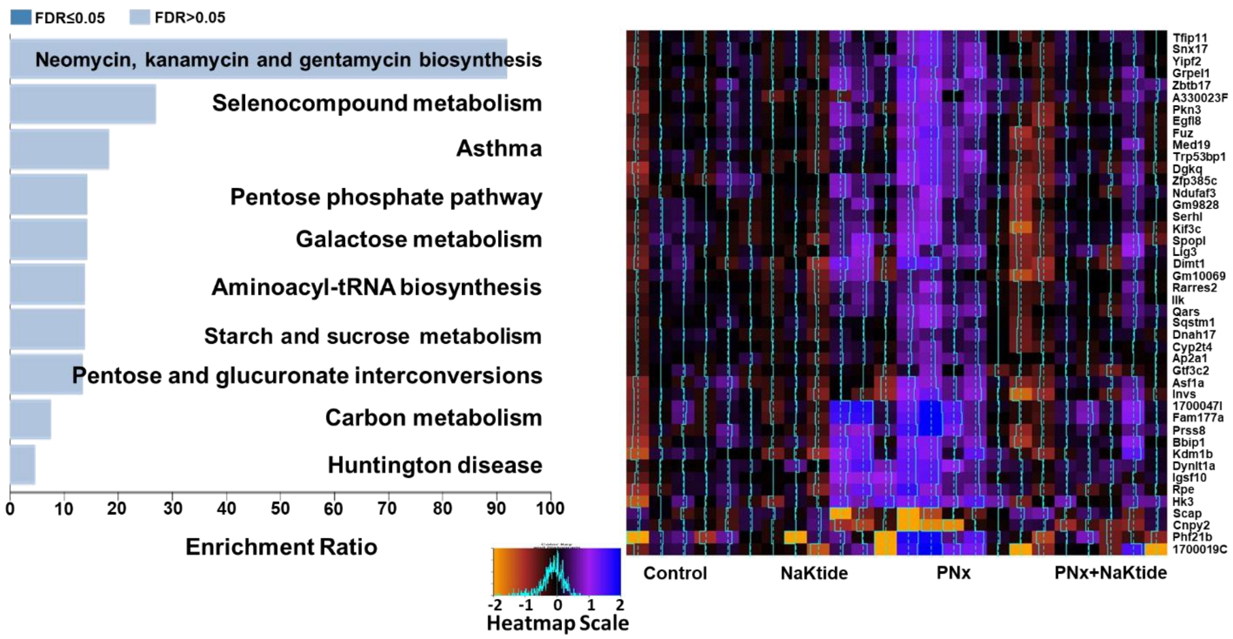

Supplemental Figure 3D: The *in vivo* KEGG ORA and the associated heat maps of GSEA scores. The figure shows the *in vivo* KEGG ORA (left panel) and the associated heat maps of GSEA scores (right panel) for groups of gene expression corresponding to the specific trait, ejection fraction. The data shows significant dysregulation of genes associated with the trait under PNx condition and the possible reversal by NaKtide treatment.

GSEA Analysis and Heat Map Data from Sienna 3 Module

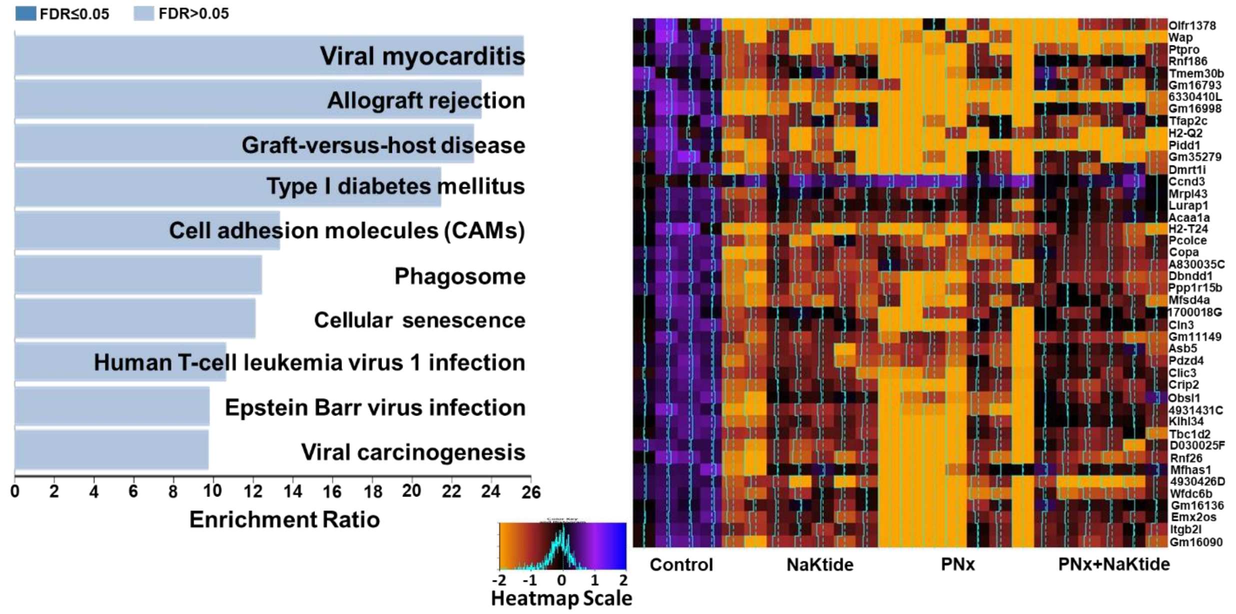

Supplemental Figure 3E: The *in vivo* KEGG ORA and the associated heat maps of GSEA scores. The figure shows the *in vivo* KEGG ORA (left panel) and the associated heat maps of GSEA scores (right panel) for groups of gene expression corresponding to the specific trait, relative wall thickness. The data shows significant dysregulation of genes associated with the trait under PNx condition and the possible reversal by NaKtide treatment.

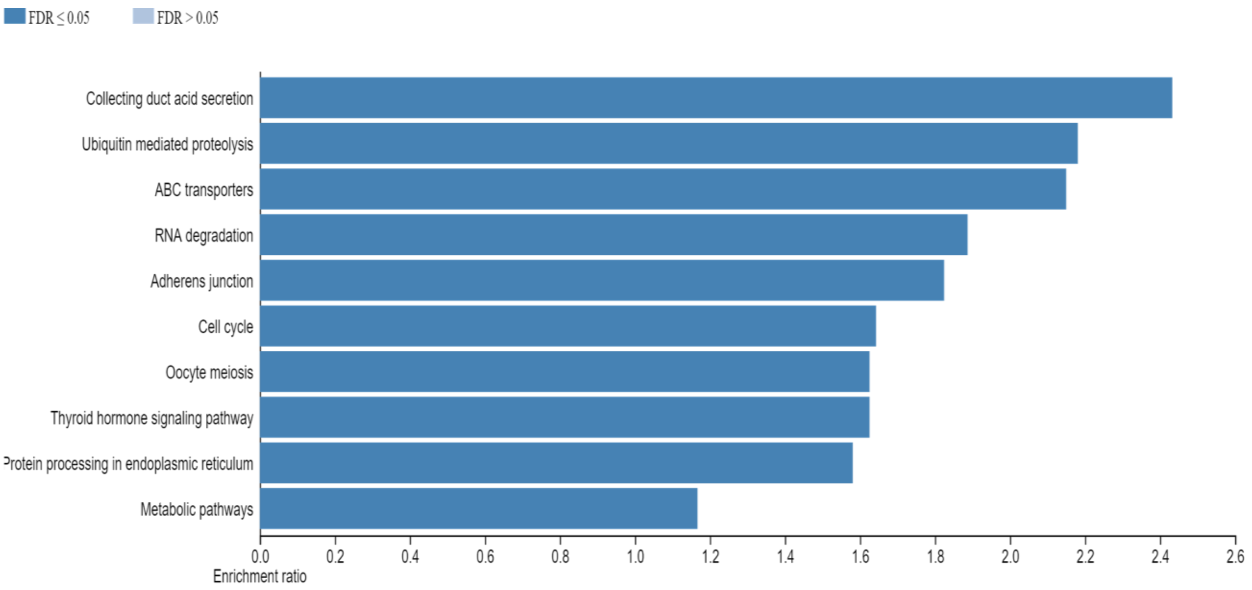

Supplementary Figure 4A: Functional enrichment analyses using Kyoto Encyclopedia of Genes and Genomes (KEGG) pathways in oxLDL treated murine adipocytes. Over expression analysis of KEGG pathways, performed on the entire data set, with the highest differentially expressed genes.

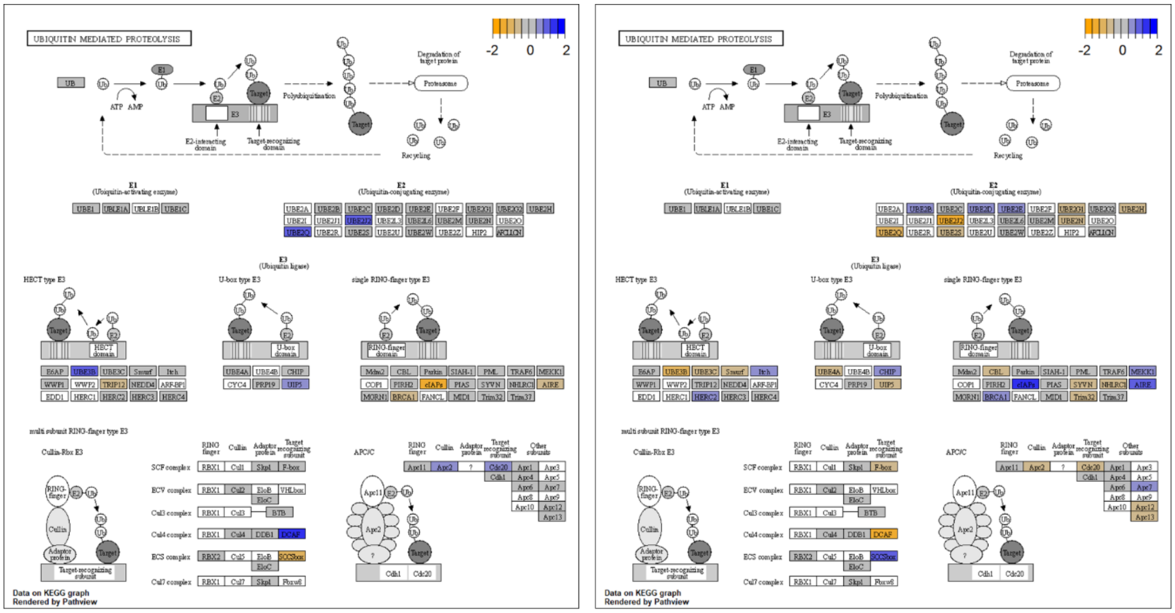

oxLDL

oxLDL+pNaKtide

Supplementary Figure 4B: KEGG pathway analysis in adipocytes exposed to oxLDL with alteration of key genes associated with oxidative stress. Genes upregulated or downregulated in pathway associated with ubiquitin mediated proteolysis. Blue represent genes upregulated and orange represent genes downregulated.

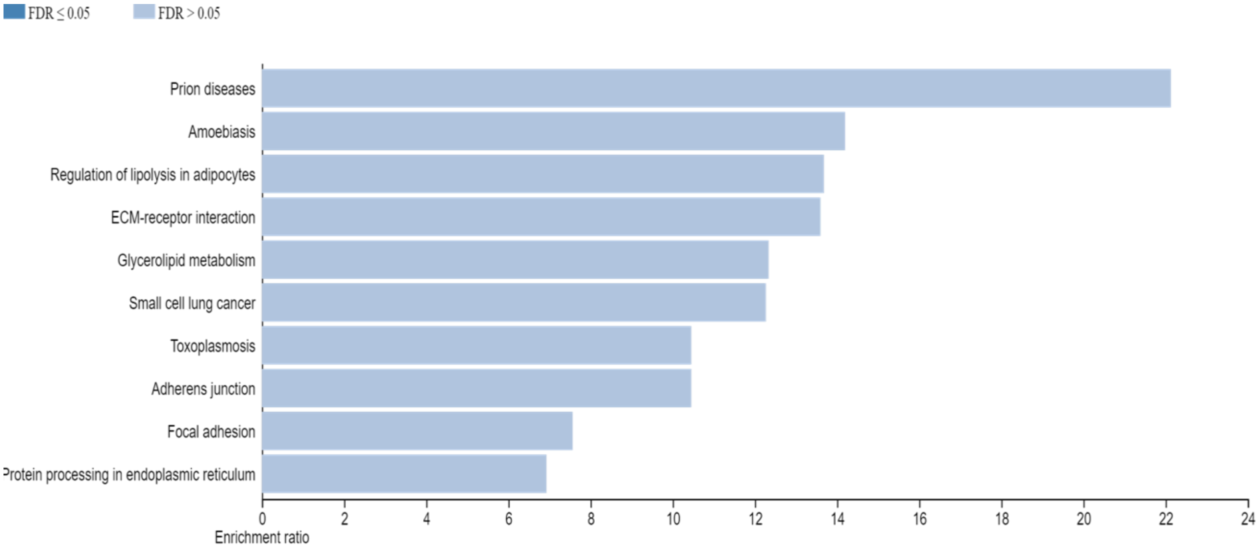

Supplementary Figure 5A: Functional enrichment analyses using Kyoto Encyclopedia of Genes and Genomes (KEGG) pathways in IS treated murine adipocytes. Over expression analysis of KEGG pathways, performed on the entire data set, with the highest differentially expressed genes.

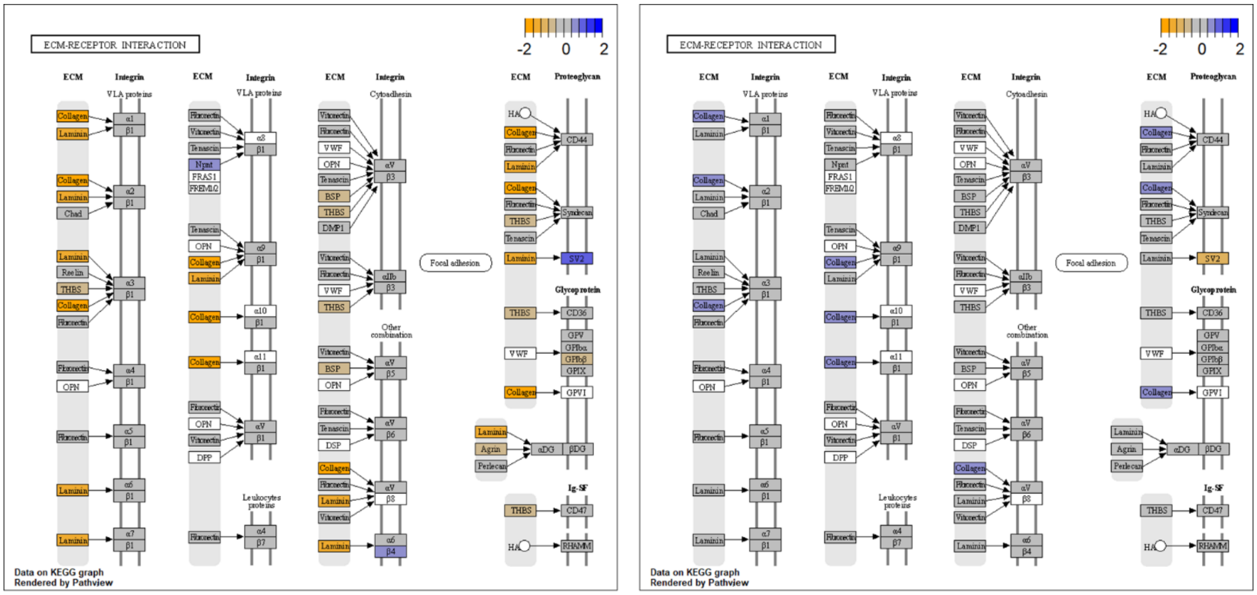

IS **IS+pNaKtide**

Supplementary Figure 5B: KEGG pathway analysis in adipocytes exposed to IS with alteration of key genes associated with oxidative stress. Genes upregulated or downregulated in pathway associated with ECM-receptor interaction. Blue represent genes upregulated and orange represent genes downregulated.

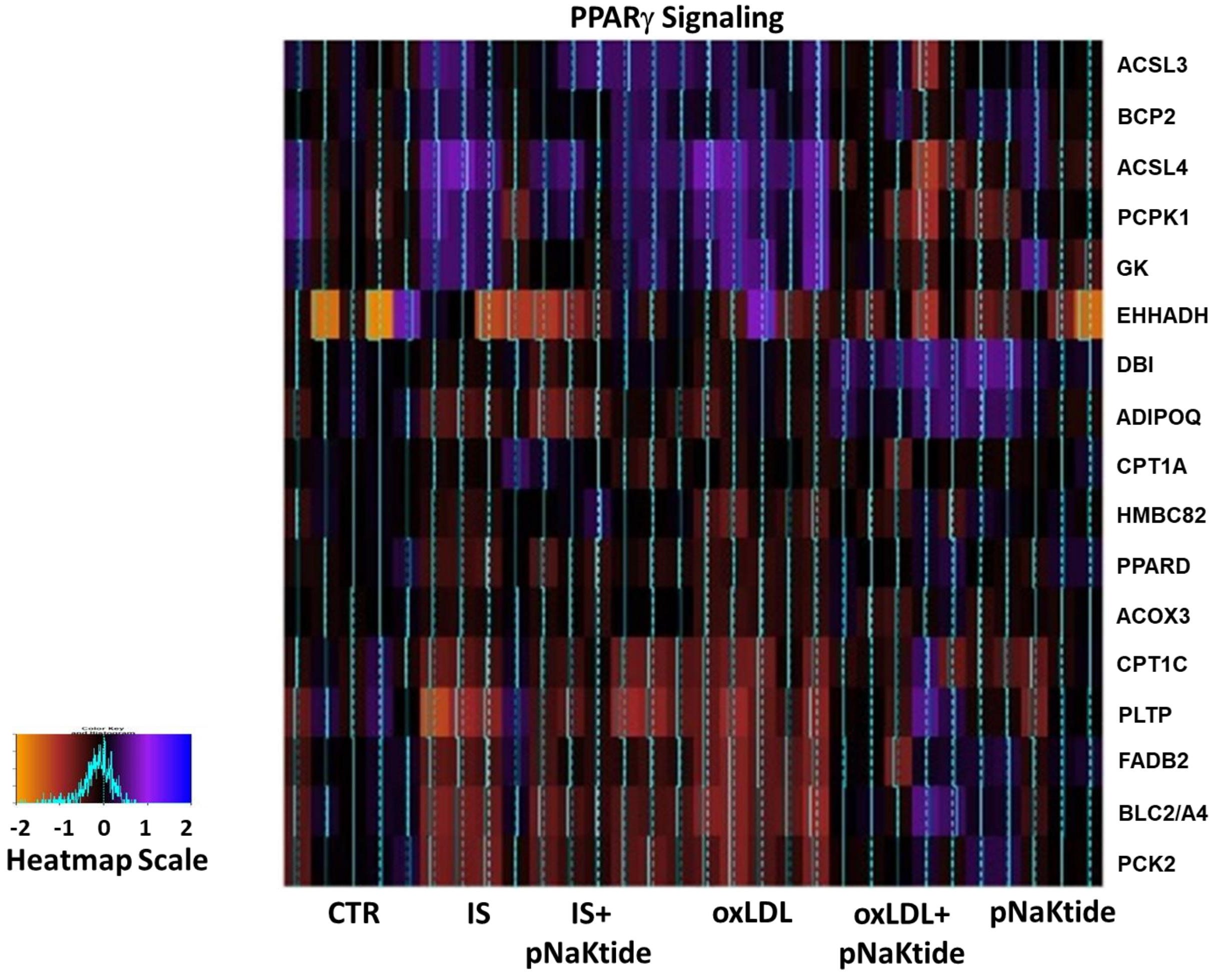

Supplementary Figure 6A: Heat maps of gene expression associated with PPAR γ signaling by OxLDL and IS using GSEA. Color coding based on log2FC with legend shown above. In the case of these pathways, differences amongst IS and oxLDL appear more likely noise in the system rather than systematic differences.

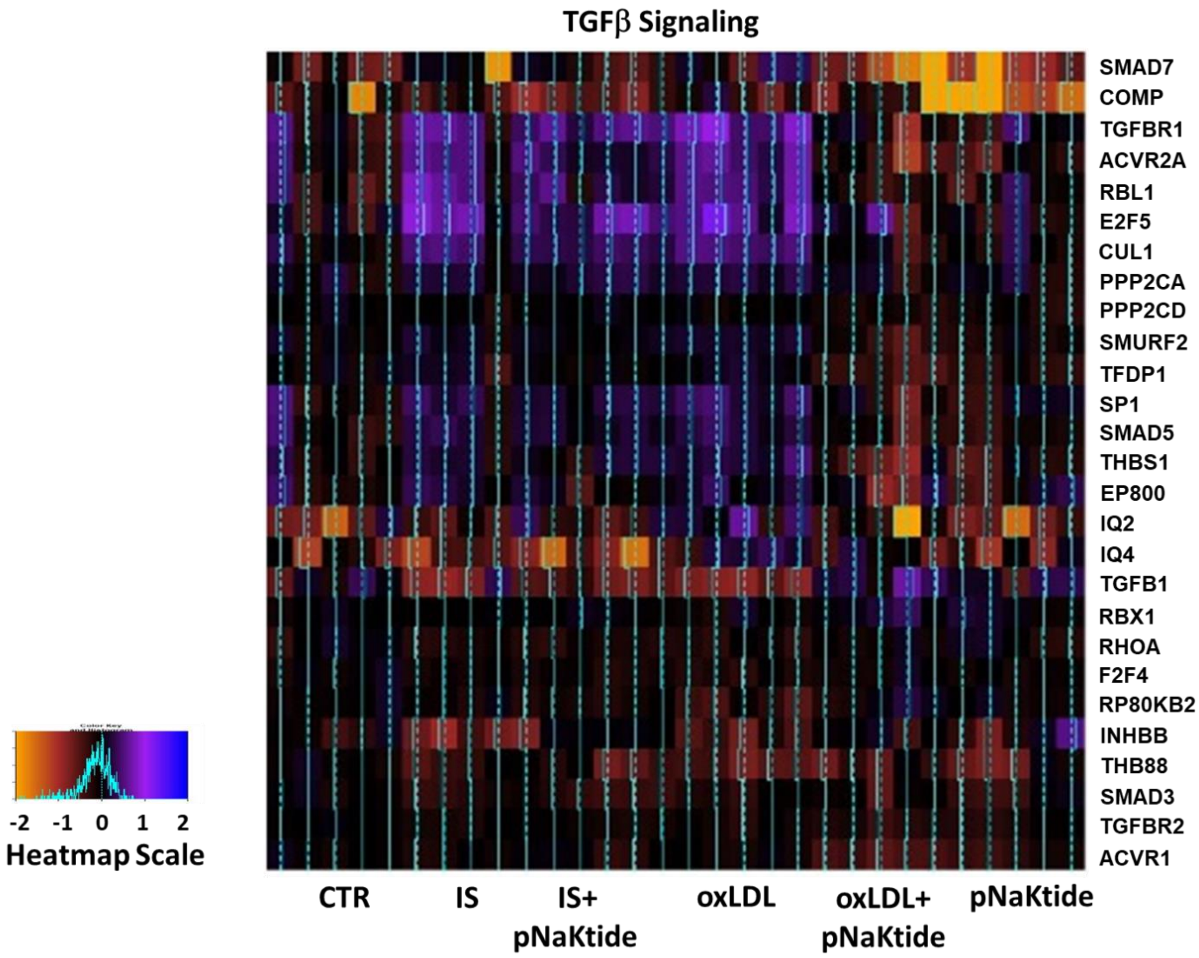

Supplementary Figure 6B: Heat maps of gene expression associated with TGFβ signaling by OxLDL and IS using GSEA. Color coding based on log2FC with legend shown above. In the case of these pathways, differences amongst IS and oxLDL appear more likely noise in the system rather than systematic differences.

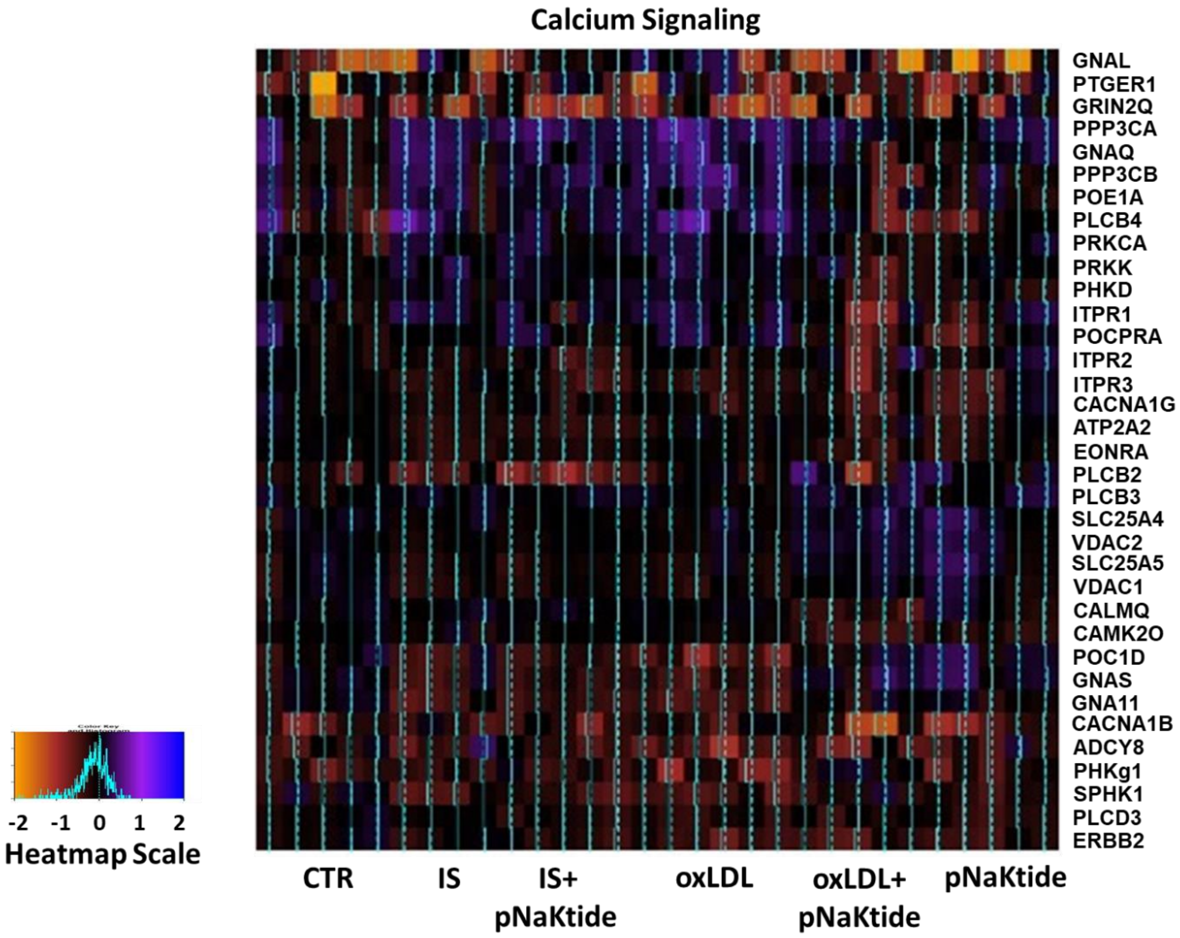

Supplementary Figure 6C: Heat maps of gene expression associated with Calcium signaling by OxLDL and IS using GSEA. Color coding based on log2FC with legend shown above. In the case of these pathways, differences amongst IS and oxLDL appear more likely noise in the system rather than systematic differences.

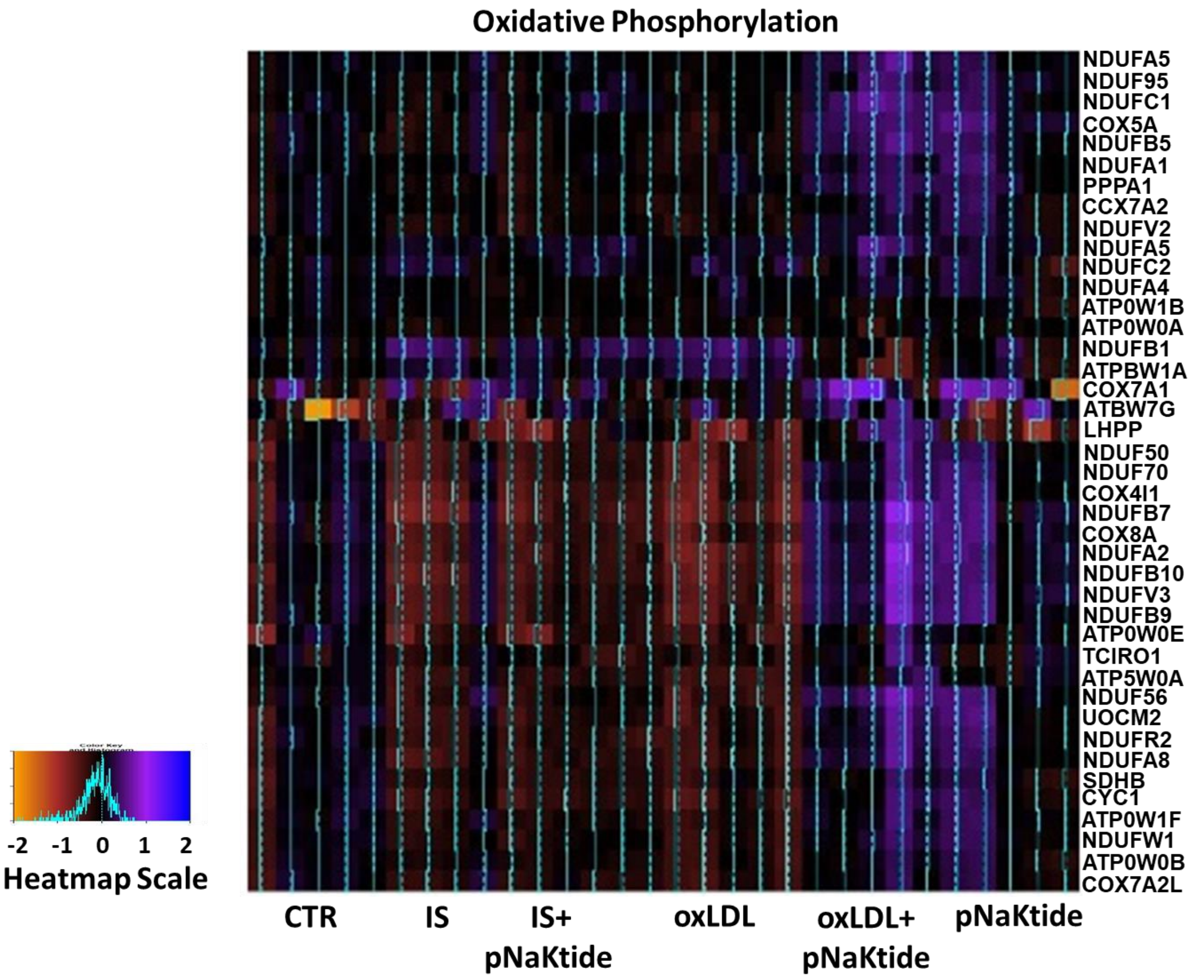

Supplementary Figure 6D: Heat maps of gene expression associated with oxidative phosphorylation by OxLDL and IS using GSEA. Color coding based on log2FC with legend shown above. In the case of these pathways, differences amongst IS and oxLDL appear more likely noise in the system rather than systematic differences.

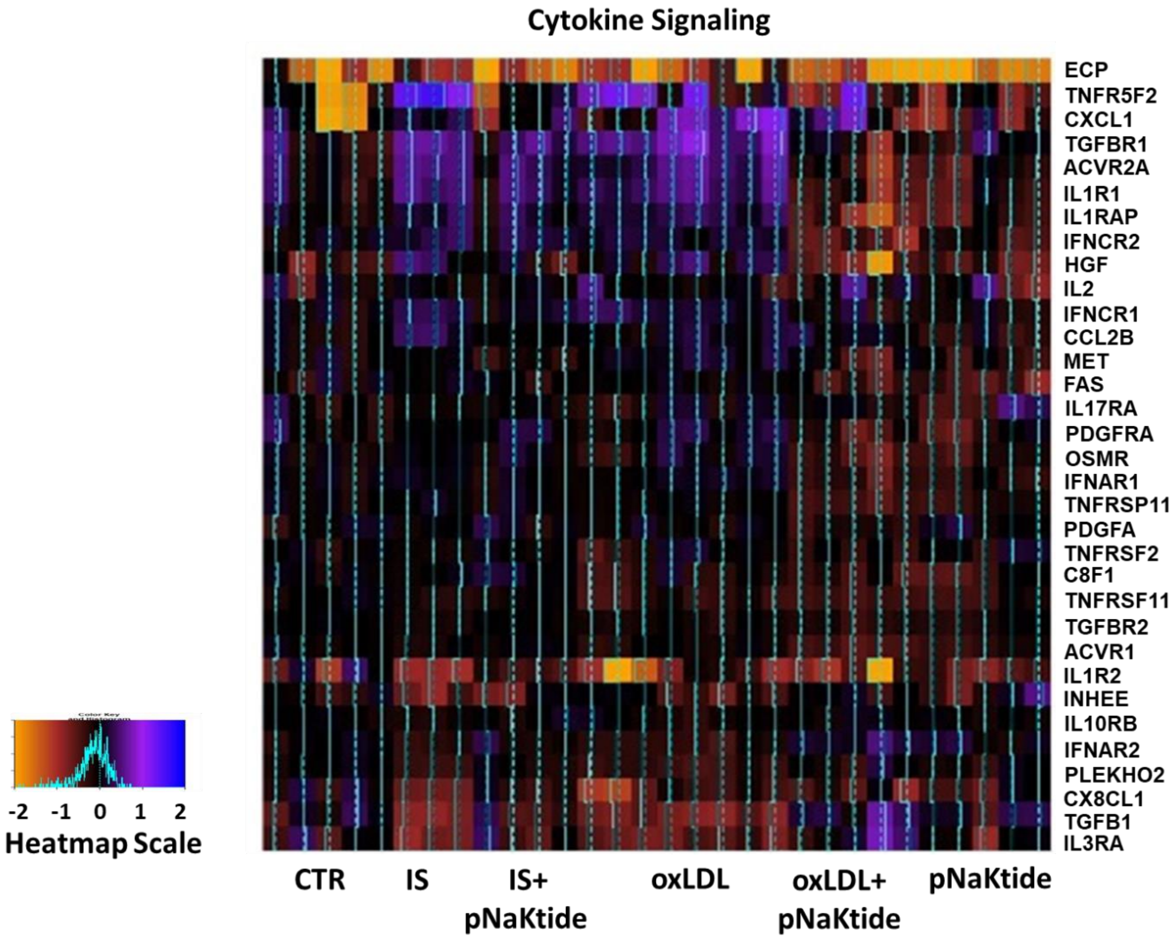

Supplementary Figure 6E: Heat maps of gene expression associated with Cytokine signaling by OxLDL and IS using GSEA. Color coding based on log2FC with legend shown above. In the case of these pathways, differences amongst IS and oxLDL appear more likely noise in the system rather than systematic differences.

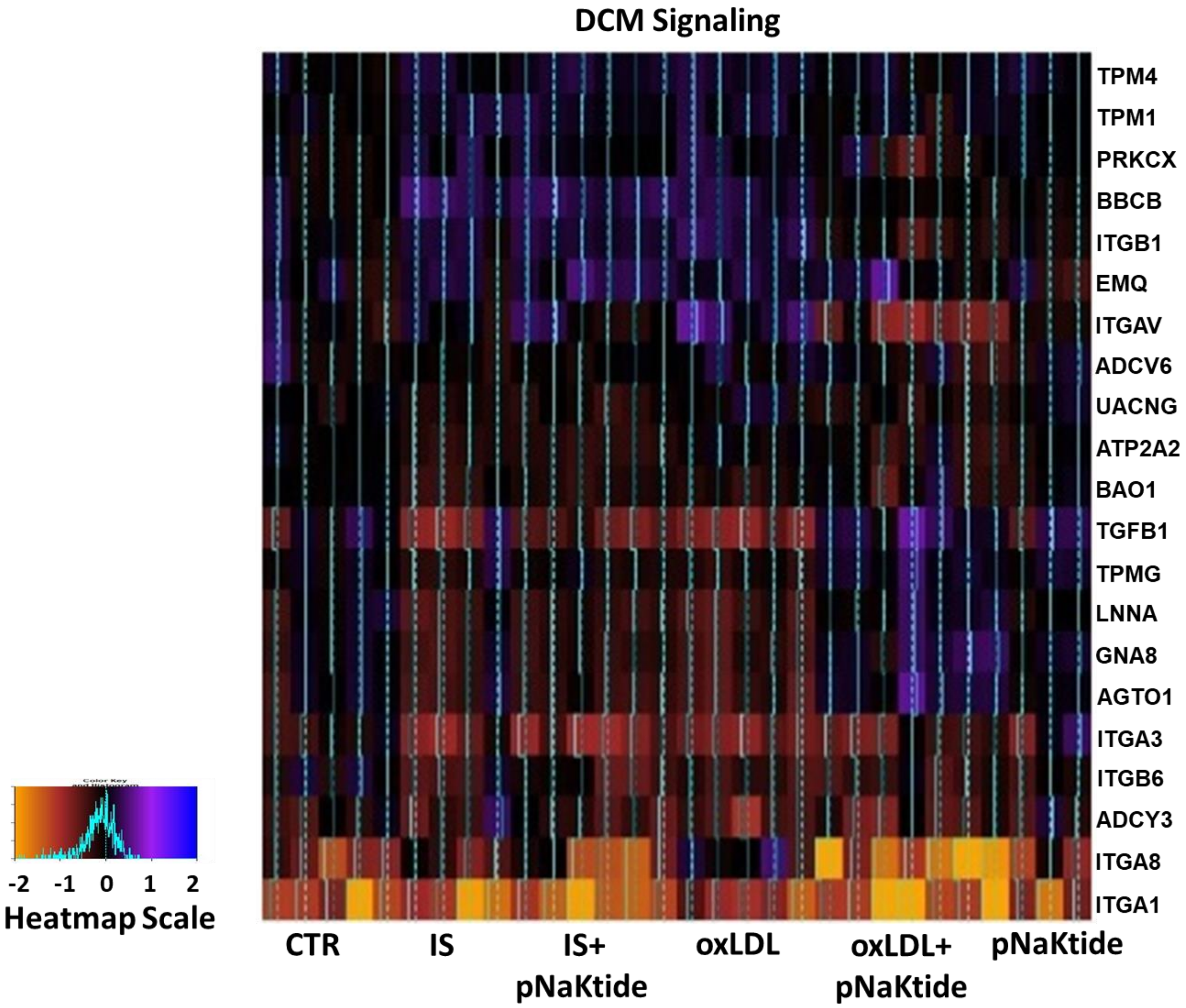

Supplementary Figure 6F: Heat maps of gene expression associated with Dilated Cardiomyopathy (DCM) signaling by OxLDL and IS using GSEA. Color coding based on log2FC with legend shown above. In the case of these pathways, differences amongst IS and oxLDL appear more likely noise in the system rather than systematic differences.

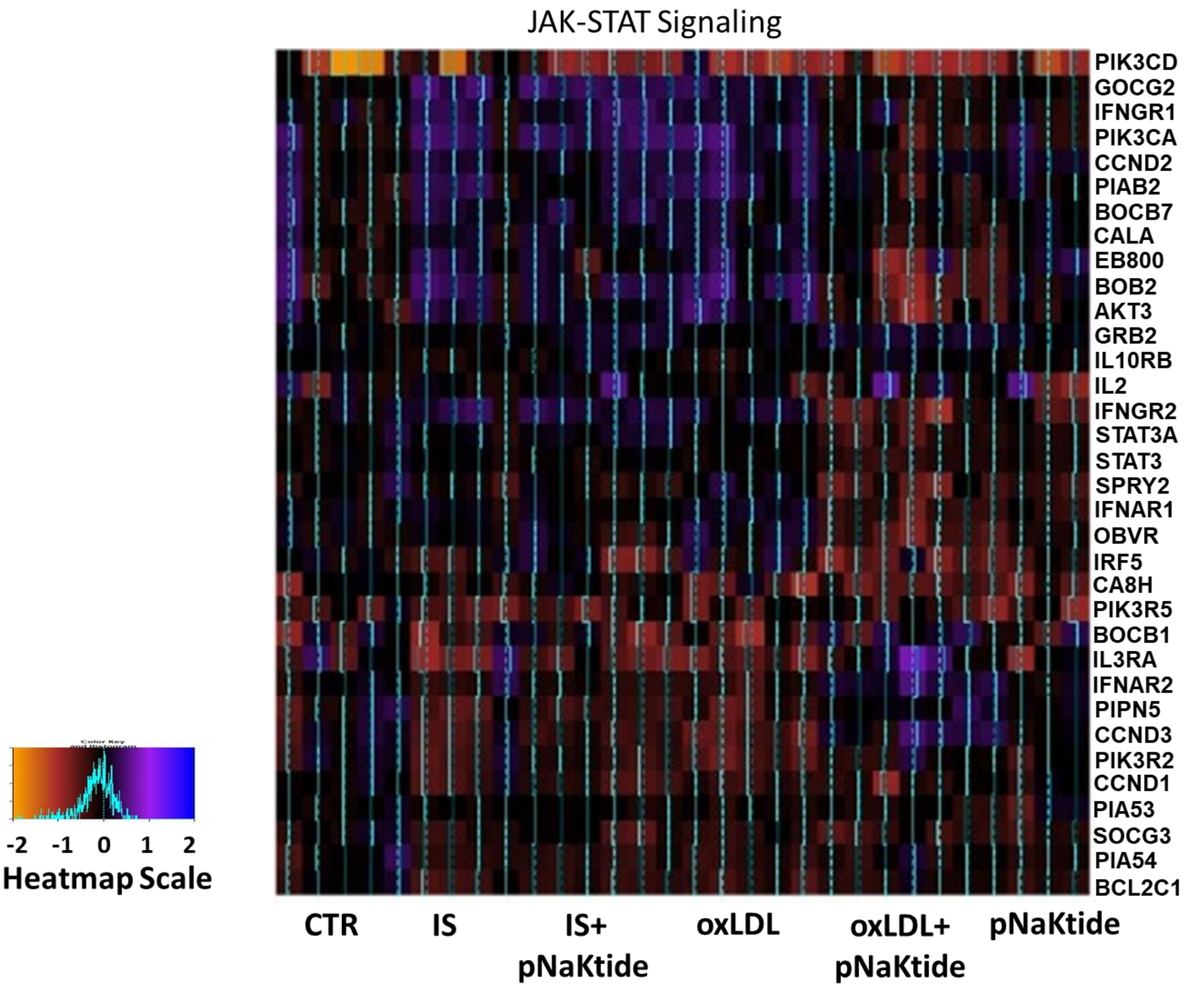

Supplementary Figure 6G: Heat maps of gene expression associated with JAK-STAT signaling by OxLDL and IS using GSEA. Color coding based on log2FC with legend shown above. In the case of these pathways, differences amongst IS and oxLDL appear more likely noise in the system rather than systematic differences.

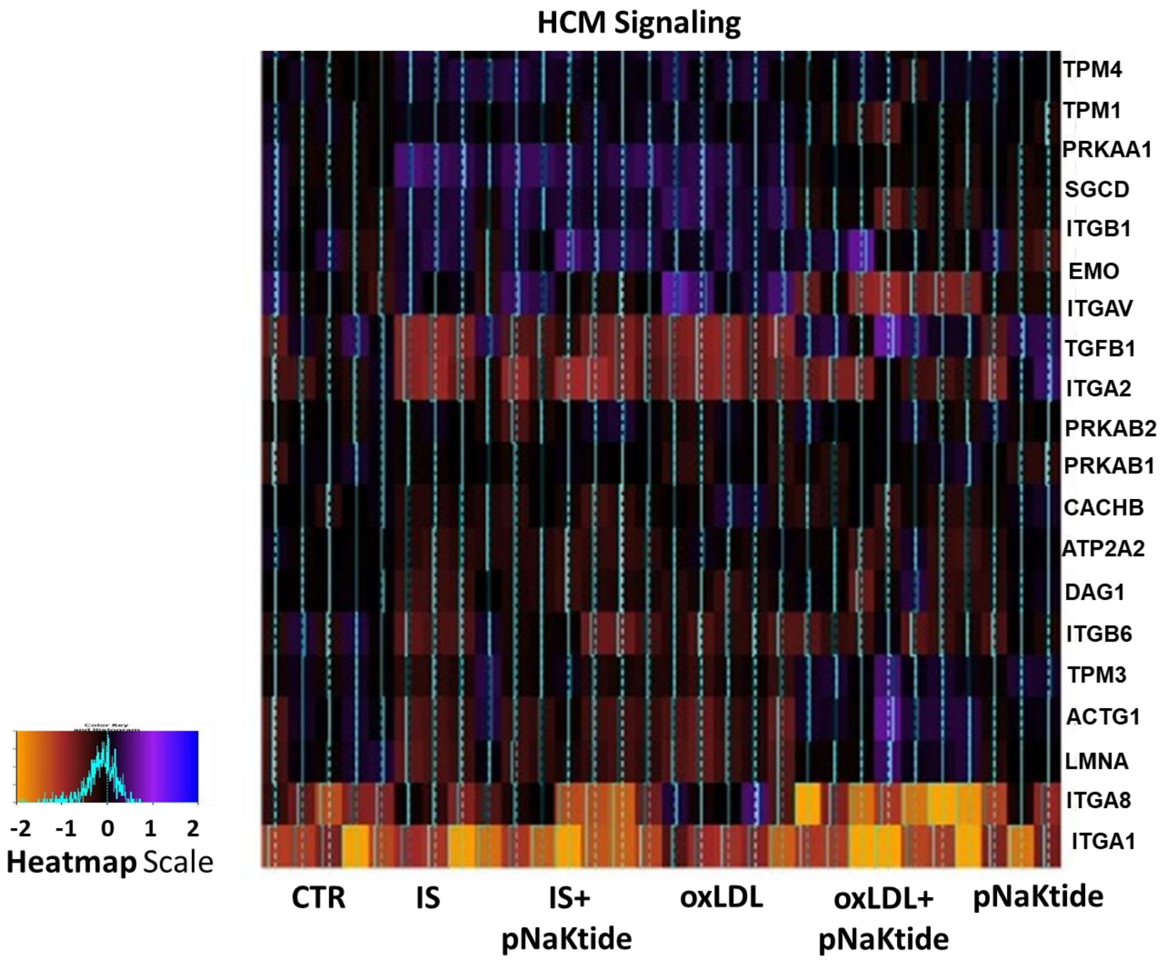

Supplementary Figure 6H: Heat maps of gene expression associated with Hypertrophic Cardiomyopathy (HCM) signaling by OxLDL and IS using GSEA. Color coding based on log2FC with legend shown above. In the case of these pathways, differences amongst IS and oxLDL appear more likely noise in the system rather than systematic differences.

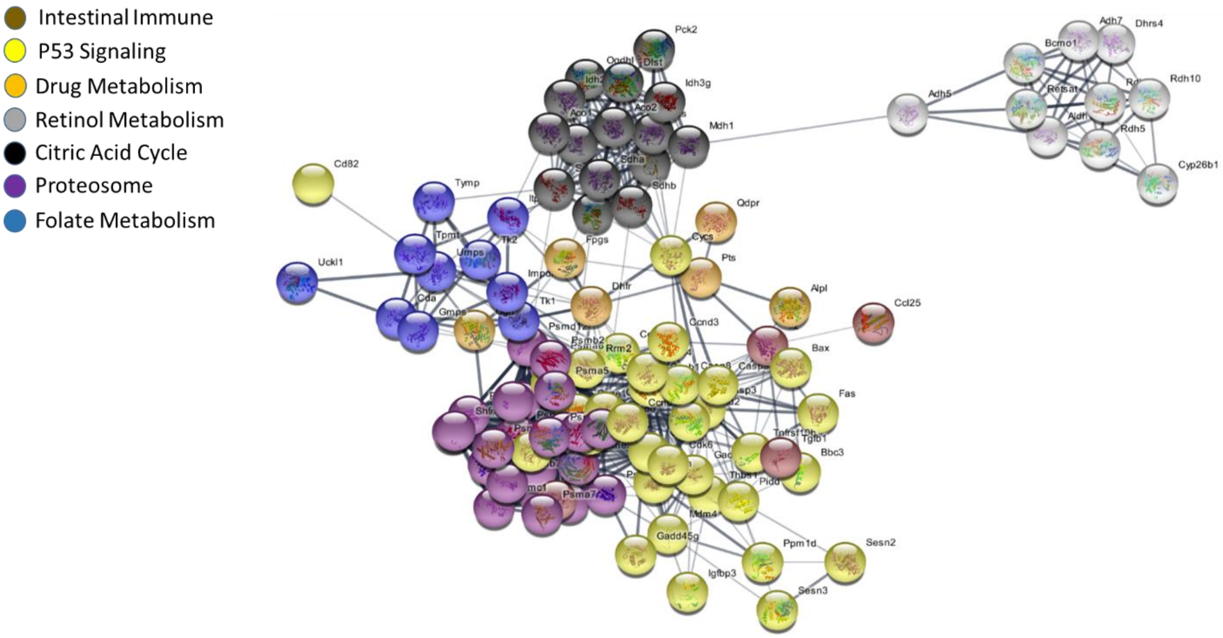

Supplementary Figure 7A: Network diagram associated with major pathways by GSEA altered *in vitro*. Color coding based on log2FC with legend shown above. All genes were identified in KEGG pathways and are therefore associated with other genes in the STRING database.

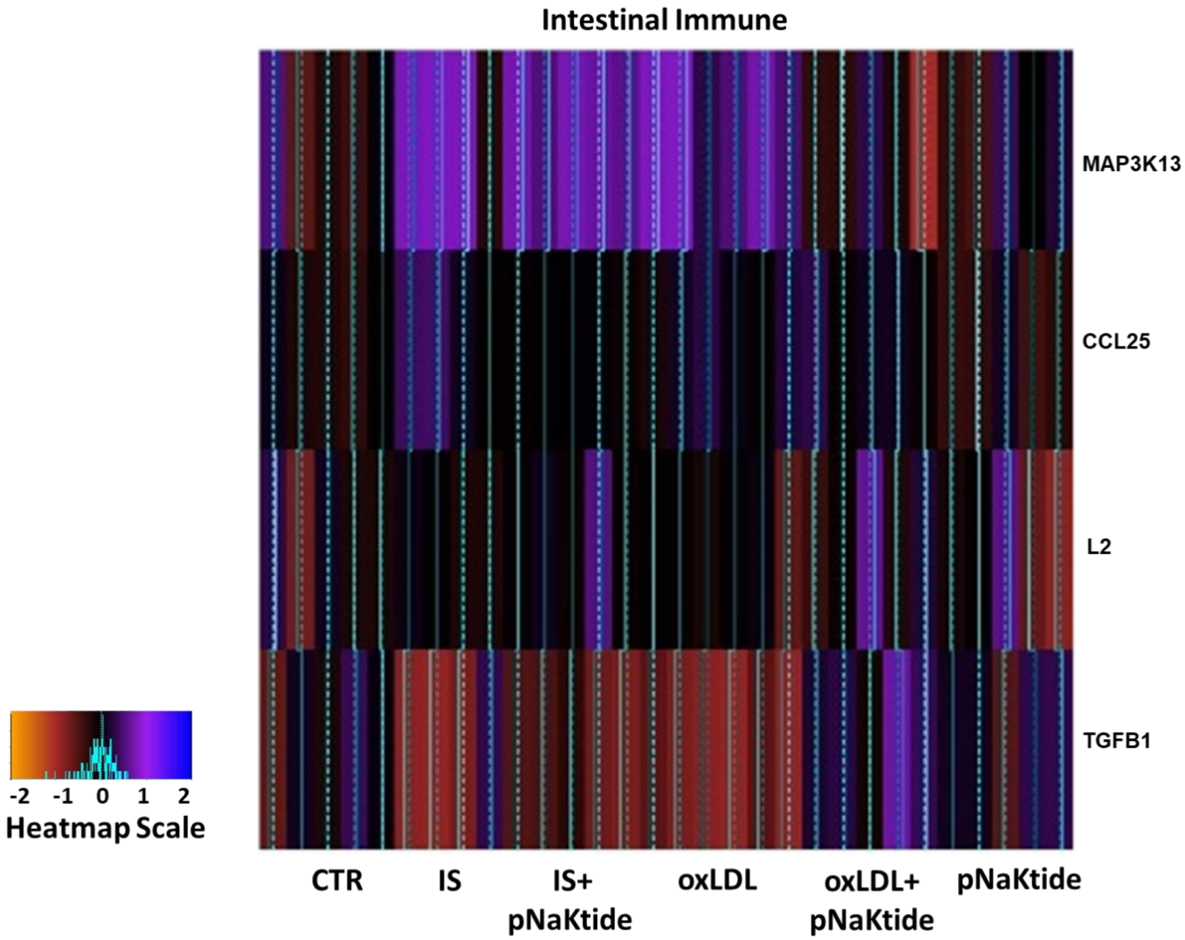

Supplementary Figure 7B: Heat map of gene expression associated with intestinal immune signaling by OxLDL and IS using GSEA. Color coding based on log2FC with legend shown above. In these case of these pathways, differences amongst IS and oxLDL appear more likely noise in the system rather than systematic differences.

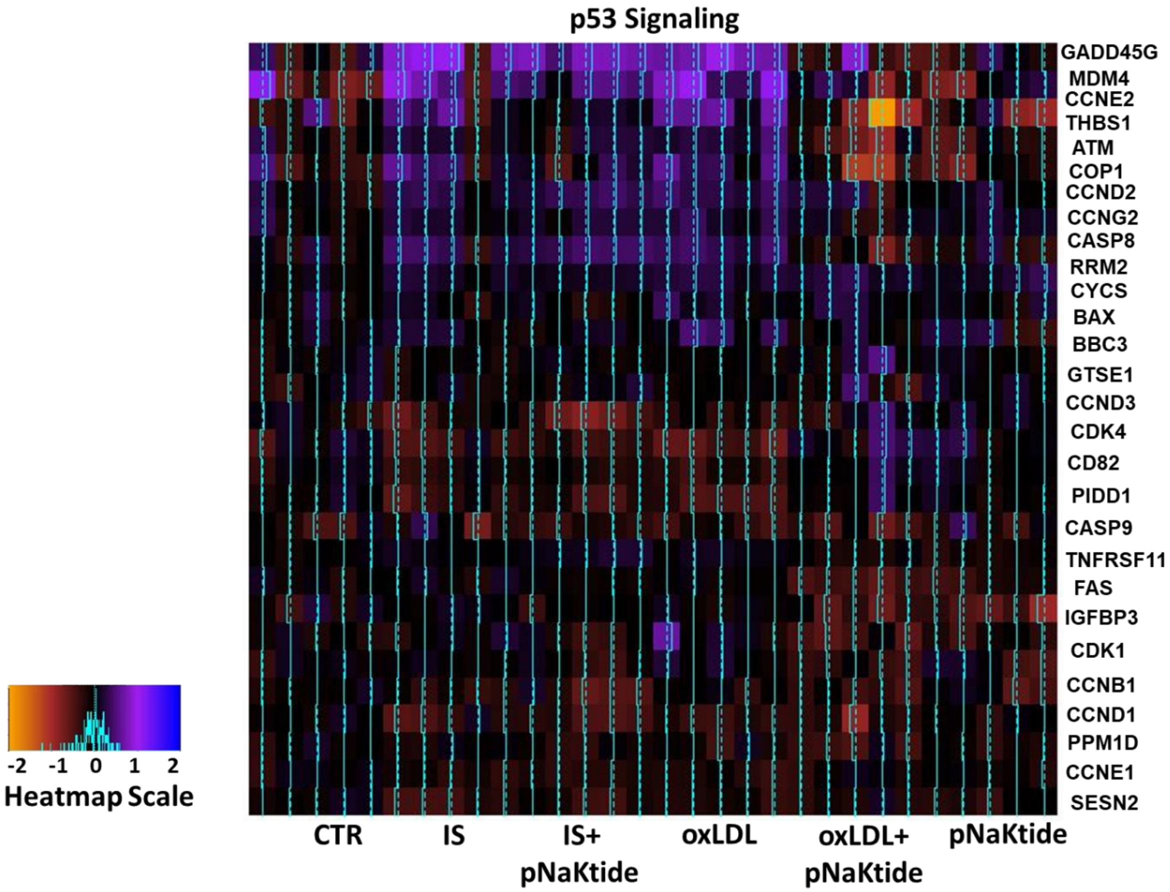

Supplementary Figure 7C: Heat map of gene expression associated with p53 signaling by OxLDL and IS using GSEA. Color coding based on log2FC with legend shown above. In these case of these pathways, differences amongst IS and oxLDL appear more likely noise in the system rather than systematic differences.

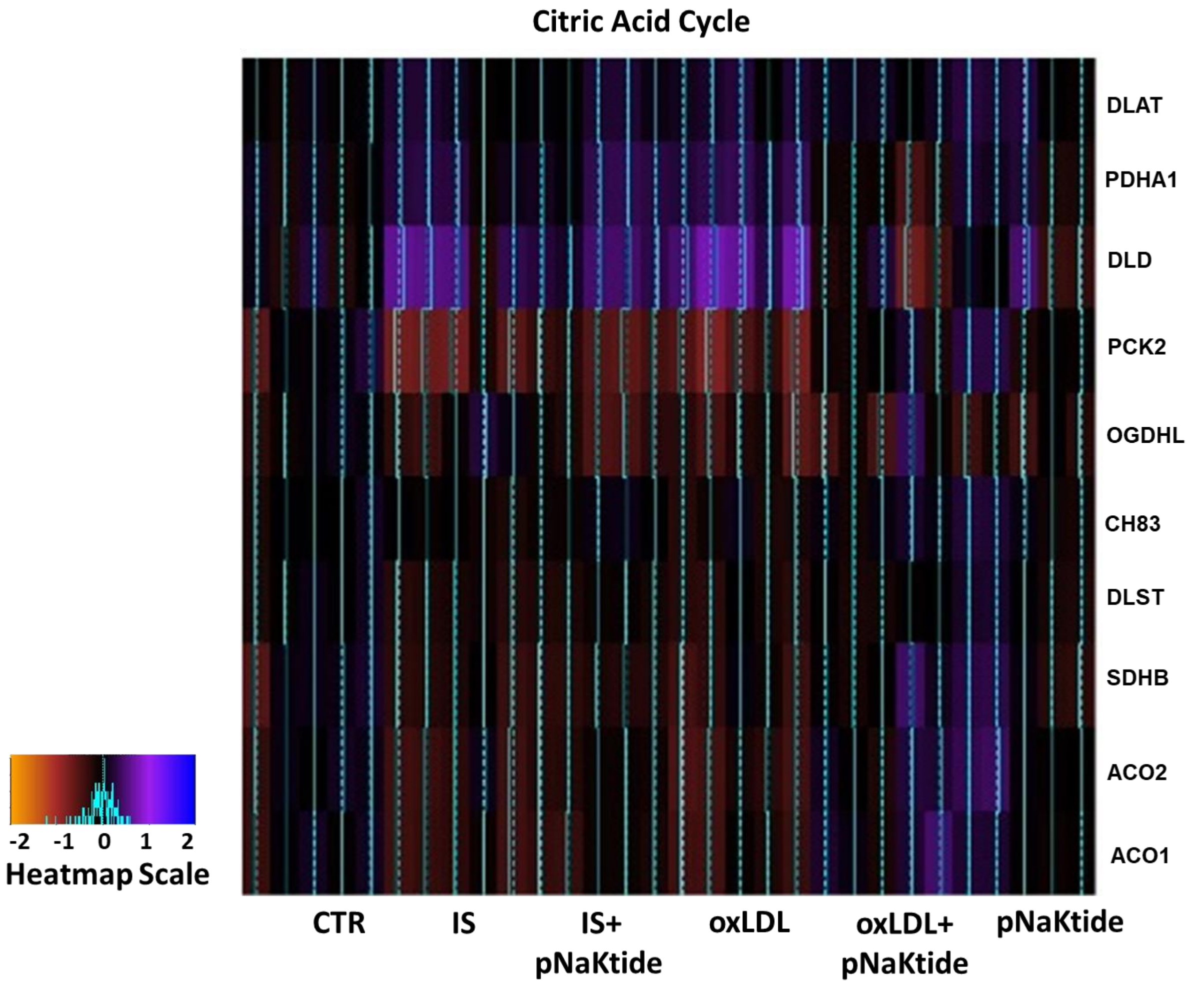

Supplementary Figure 7D: Heat map of gene expression associated with citric acid cycle by OxLDL and IS using GSEA. Color coding based on log2FC with legend shown above. In these case of these pathways, differences amongst IS and oxLDL appear more likely noise in the system rather than systematic differences.

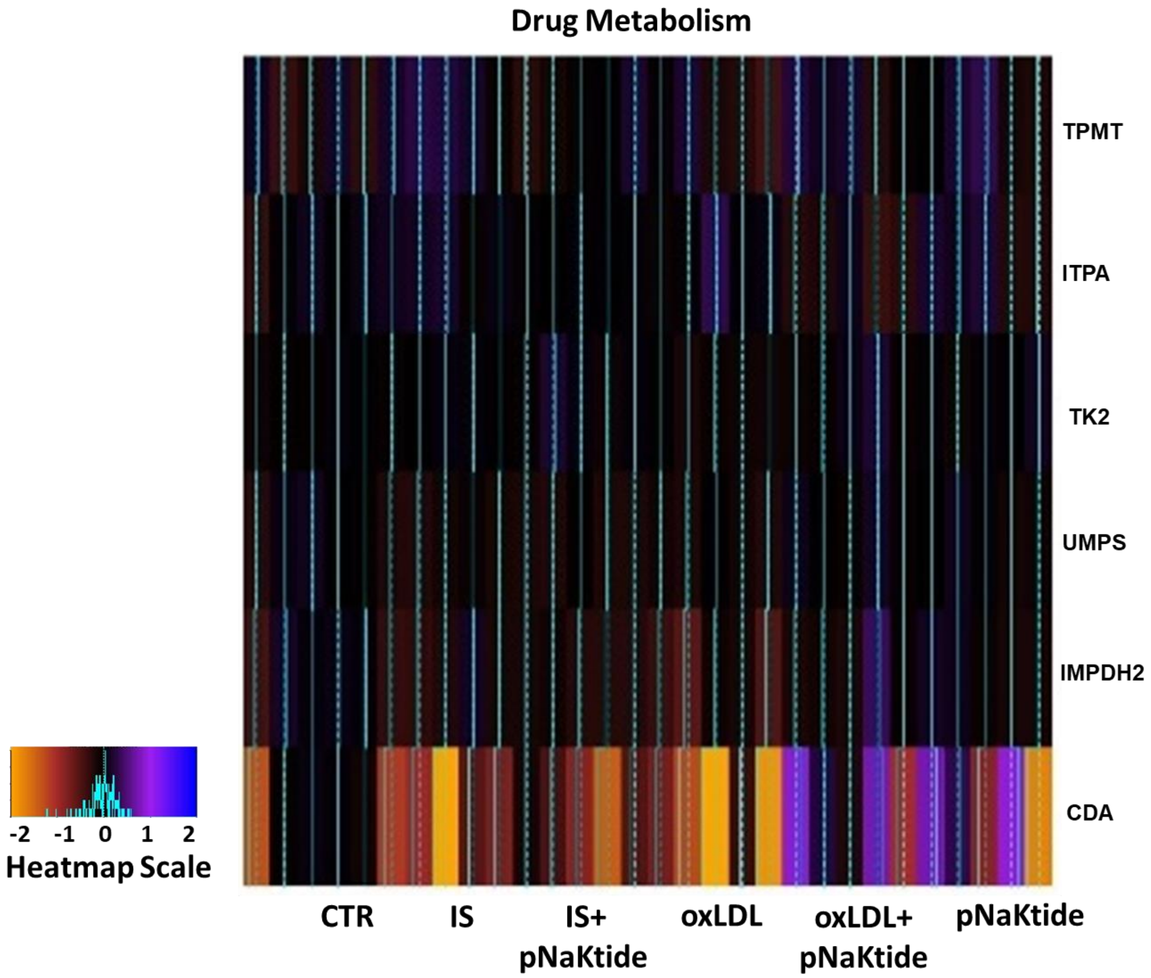

Supplementary Figure 7E: Heat map of gene expression associated with drug metabolism by OxLDL and IS using GSEA. Color coding based on log2FC with legend shown above. In these case of these pathways, differences amongst IS and oxLDL appear more likely noise in the system rather than systematic differences.

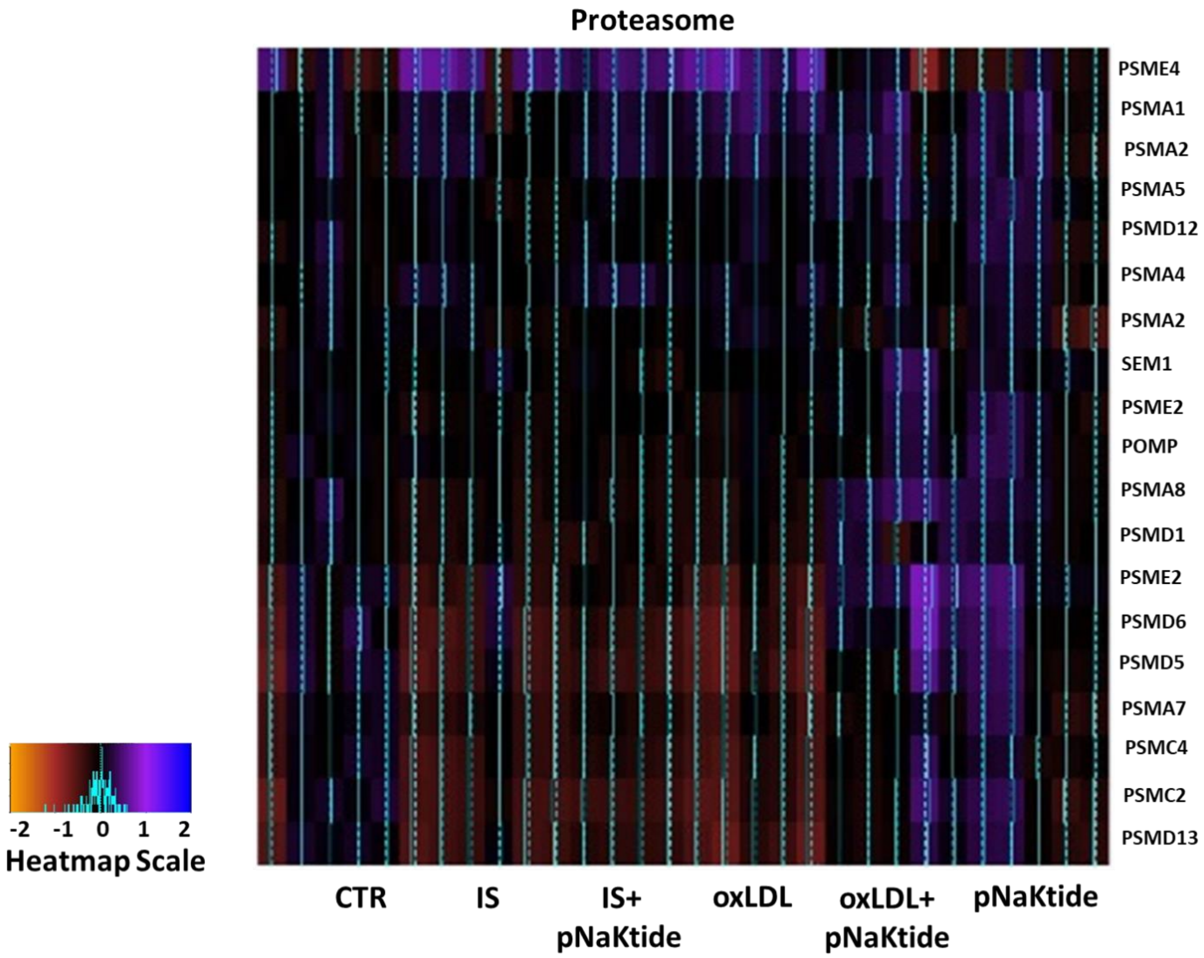

Supplementary Figure 7F: Heat map of gene expression associated with proteasome signaling by OxLDL and IS using GSEA. Color coding based on log2FC with legend shown above. In these case of these pathways, differences amongst IS and oxLDL appear more likely noise in the system rather than systematic differences.

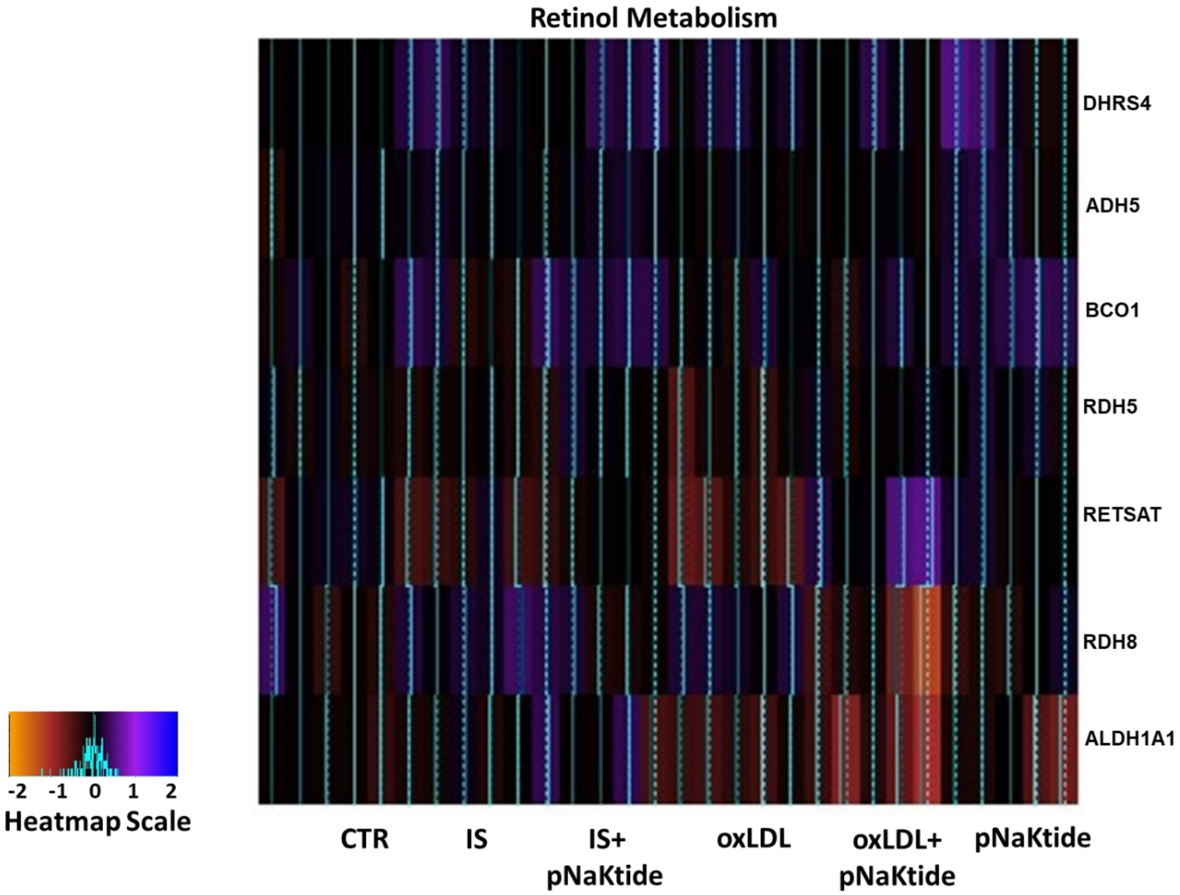

Supplementary Figure 7G: Heat map of gene expression associated with retinol metabolism by OxLDL and IS using GSEA. Color coding based on log2FC with legend shown above. In these case of these pathways, differences amongst IS and oxLDL appear more likely noise in the system rather than systematic differences.

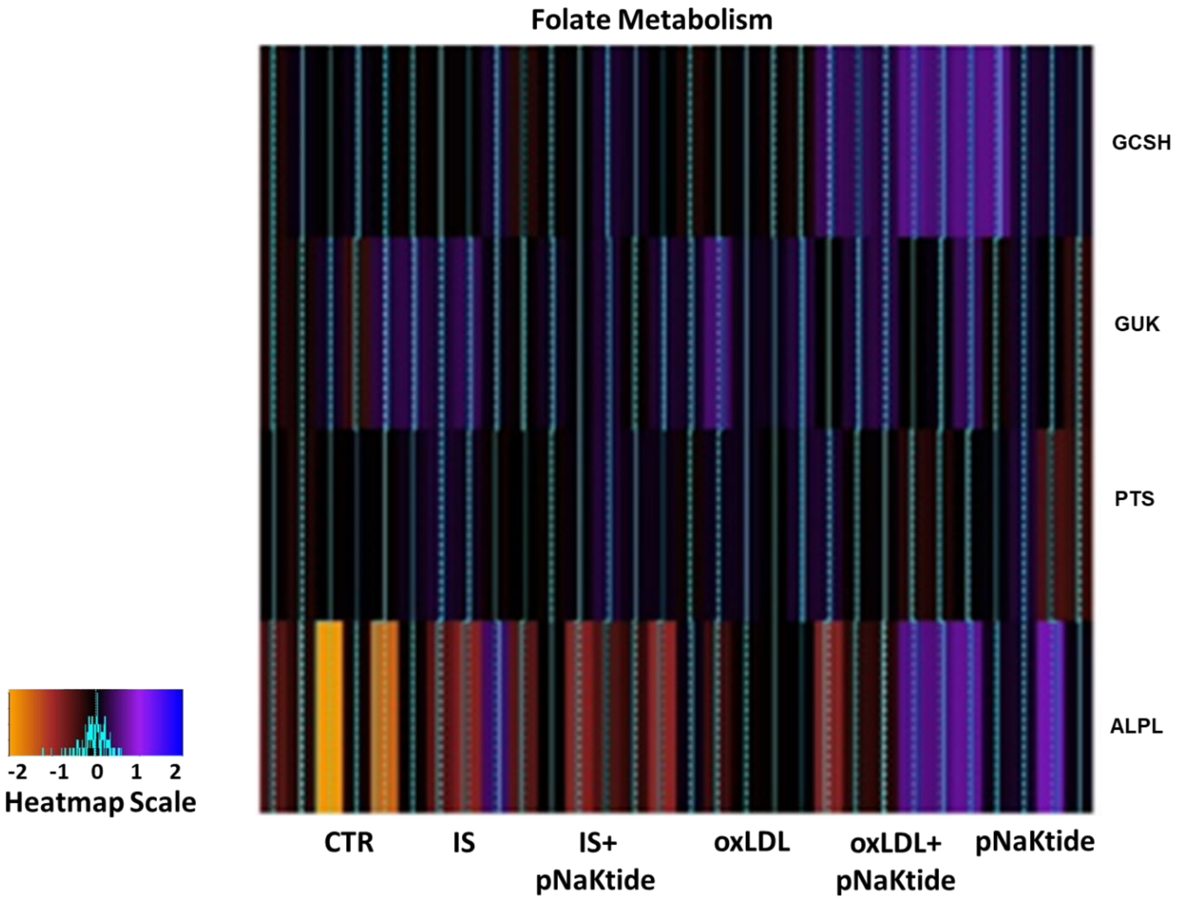

Supplementary Figure 7H: Heat map of gene expression associated with folate metabolism by OxLDL and IS using GSEA. Color coding based on log2FC with legend shown above. In these case of these pathways, differences amongst IS and oxLDL appear more likely noise in the system rather than systematic differences.

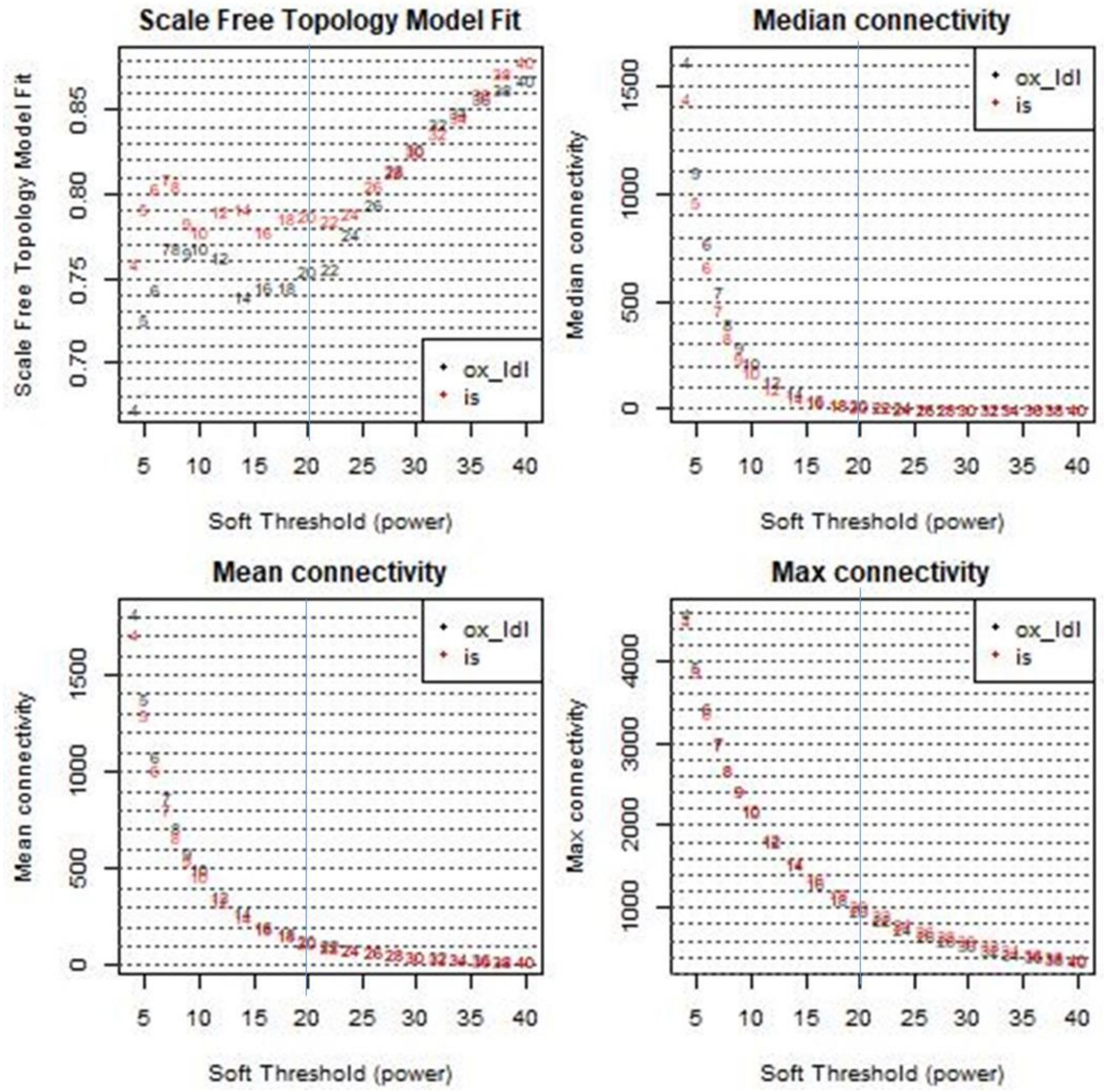

Supplementary Figure 8. Scale independence and Mean connectivity as a function of Soft Threshold in *in vitro* datasets. Based on these data, we continued to employ a soft threshold (power) of 20 to construct the network(s) described subsequently in the manuscript.
